# ﻿Seven new species of *Alternaria* (Pleosporales, Pleosporaceae) associated with Chinese fir, based on morphological and molecular evidence

**DOI:** 10.3897/mycokeys.101.115370

**Published:** 2024-01-05

**Authors:** Jiao He, De-Wei Li, Wen-Li Cui, Lin Huang

**Affiliations:** 1 Co-Innovation Center for Sustainable Forestry in Southern China, Nanjing Forestry University, Nanjing, Jiangsu 210037, China Nanjing Forestry University Nanjing China; 2 The Connecticut Agricultural Experiment Station Valley Laboratory, Windsor, CT 06095, USA The Connecticut Agricultural Experiment Station Valley Laboratory Windsor United States of America

**Keywords:** *Alternaria*, *Cunninghamialanceolata*, diversity, leaf blight, new species, pathogenicity

## Abstract

Chinese fir (*Cunninghamialanceolata*) is a special fast-growing commercial tree species in China and has significant ecological and economic value. However, it experienced damage from leaf blight caused by pathogenic fungi of the genus *Alternaria*. To determine the diversity of *Alternaria* species associated with leaf blight of Chinese fir in China, infected leaves were collected from five major cultivation provinces (Fujian, Henan, Hunan, Jiangsu and Shandong provinces). A total of 48 fungal strains of *Alternaria* were obtained. Comparison of morphology and phylogenetic analyses, based on nine loci (ITS, SSU, LSU, GAPDH, RPB2, TEF1, Alt a1, endoPG and OPA10-2) of the representative isolates as well as the pairwise homoplasy index tests, revealed that the fungal strains belonged to seven undescribed taxa of *Alternaria*, which are described here and named as *Alternariacunninghamiicola***sp. nov.**, *A.dongshanqiaoensis***sp. nov.**, *A.hunanensis***sp. nov.**, *A.kunyuensis***sp. nov.**, *А. longqiaoensis***sp. nov.**, *A.shandongensis***sp. nov.** and *A.xinyangensis***sp. nov.** In order to prove Koch’s postulates, pathogenicity tests on detached Chinese fir leaves revealed significant pathogenicity amongst these species, of which *A.hunanensis* is the most pathogenic to Chinese fir. This study represents the first report of *A.cunninghamiicola*, *A.dongshanqiaoensis*, *A.hunanensis*, *A.kunyuensis*, *A.longqiaoensis*, *A.shandongensis* and *A.xinyangensis* causing leaf blight on Chinese fir. Knowledge obtained in this study enhanced our understanding of *Alternaria* species causing leaf blight on Chinese fir and was crucial for the disease management and the further studies in the future.

## ﻿Introduction

*Alternaria* is a genus (Pleosporaceae, Pleosporales, Ascomycota) (Seifert et al. 2011), which originally was described in 1816 by Nees (1816), typified with *A.tenuis* Nees. Since then, more than 900 epithets and varieties/f. spp. have been published in *Alternaria* (MycoBank 2023). At present, there are over 360 species (Wijayawardene et al. 2020). *Alternaria* is a ubiquitous fungal genus that includes saprobic, endophytic and pathogenic species (Li et al. 2023). For example, *Alternaria* species have been recorded as endophytes in grasses, angiosperms, rice and other herbaceous plants and shrubs (Fisher and Petrini 1992; Schulz et al. 1993; Rosa et al. 2009; Polizzotto et al. 2012) and have been also isolated from soil (Hong and Pryor 2004). Many *Alternaria* species are saprobes on a variety of plant tissues in different habitats (Thomma 2003; Liu et al. 2015b; Wanasinghe et al. 2018). Some *Alternaria* species, such as *A.alternata*, produce host-specific toxins (Hyde et al. 2018). Several taxa are also important postharvest pathogens, for example, *A.alternata* and *A.solani* (El-Goorani and Sommer 1981; Reddy et al. 2000), or airborne fungal allergens/pathogens-causing upper respiratory tract infections and asthma in humans (Mitakakis et al. 2001; Woudenberg et al. 2015; Hyde et al. 2018). Due to the significant negative health effects of *Alternaria* on humans and their surroundings, a correct and rapid identification of *Alternaria* species would be of great significance to researchers, plant pathologists, medical mycologists, other biological professionals and the public alike (Woudenberg et al. 2013).

The taxonomy of *Alternaria* species especially small-spored species within the *alternata* species group are particularly challenging because few morphological characters are able to clearly differentiate taxa and these characters are strongly influenced by the environment. Morphological characteristics, such as colour, size, shape of conidia and sporulation patterns have been used for the identification and classification of *Alternaria* species (Simmons 1992). Wiltshire (1945) divided *Alternaria* into three major sections, Brevicatenatae, Longicatenatae and Noncatenatae, based on conidial catenation. However, this division is unreliable as some of these characters overlap amongst species and vary depending on the cultural conditions, such as temperature and substrate (Simmons and Roberts 1993). Simmons (1992, 1995) arranged several species groups within *Alternaria* based on the morphological similarity amongst species. Some other genera, such as *Stemphylium* (Wallroth, 1833) and *Ulocladium* (Preuss, 1852) also produce phaeodictyospores and are morphologically similar to *Alternaria*, and this has further led to taxonomic complications (Bigelow 2003). Simmons (2007) revised *Alternaria* taxonomy, based on morphology and 275 species were recognised. At the same time, Simmons (2007) proposed three new genera *Alternariaster*, *Chalastospora* and *Teretispora* for some species that were previously described in *Alternaria*.

However, molecular phylogeny has revealed polyphyletic taxa within *Alternaria* and *Alternaria* species clades, which do not always correlate with morphological species-groups (Inderbitzin et al. 2006; Runa et al. 2009; Lawrence et al. 2012). Pryor and Gilbertson (2000) elucidated relationships amongst *Alternaria*, *Stemphylium* and *Ulocladium* based on ITS and SSU sequence data and revealed that *Stemphylium* species were phylogenetically distinct from *Alternaria* and *Ulocladium* species. Most *Alternaria* and *Ulocladium* clustered together in a large *Alternaria*/*Ulocladium* clade (Pryor and Gilbertson 2000). Chou and Wu (2002) confirmed that filament-beaked *Alternaria* species constitute a monophyletic group distinct from the other members in this genus and hypothesised that this group is evolutionarily distinct, based on phylogenies of ITS sequence. Two new species groups, *A.panax* and *A.gypsophilae* were introduced by Lawrence et al. (2013) with phylogenetic evidence and they accepted eight well supported asexual species-sections within *Alternaria*, while the taxa with known sexual morphs, the *A.infectoria* species-groups, were not given the similar rank. Woudenberg et al. (2013) delineated taxa within *Alternaria* and allied genera, based on SSU, LSU, ITS, GAPDH, RPB2 and TEF1 sequence data. The generic circumscription of *Alternaria* was emended and 24 internal clades in the *Alternaria* complex were treated as sections, together with six monotypic lineages (Woudenberg et al. 2013; Gannibal et al. 2022). Woudenberg et al. (2013) also demoted the genera *Allewia*, *Brachycladium*, *Chalastospora*, *Chmelia*, *Crivellia*, *Embellisia*, *Lewia*, *Nimbya*, *Sinomyces*, *Teretispora*, *Ulocladium*, *Undifilum* and *Ybotromyces* to synonymy with *Alternaria*. Therefore, the use of DNA sequence data is very important in resolving *Alternaria* taxonomy.

The DNA-based classification of the genus *Alternaria* has, so far, relied on over ten gene/region loci, including the nuclear small subunit (SSU) rRNA, large subunit (LSU) rRNA, internal transcribed spacer (ITS), glyceraldehyde-3-phosphate dehydrogenase (GAPDH), RNA polymerase II 2^nd^ largest subunit (RPB2), translation elongation factor 1-α (TEF1), *Alternaria* major allergen (Alt a1), endopolygalacturonase (endoPG), anonymous gene region (OPA10-2), calmodulin (CAL) and eukaryotic orthologous group (KOG) (Lawrence et al. 2013; Woudenberg et al. 2013; Woudenberg et al. 2015; Ghafri et al. 2019; Jayawardena et al. 2019a, 2019b). Several studies have shown that multilocus phylogenetic identification can classify or segregate *Alternaria* species. For instance, Li et al. (2023) used sequences of ITS, *LSU*, *TEF1*, *RPB2*, *GAPDH* and Alt a1 loci and described 18 new species in sect. Alternaria, sect. Infectoriae, sect. Porri and sect. Radicina. Aung et al. (2020) reported the first case of small-spored *A.alternata* associated with Koerle pear (Pyrus×sinkiangensis T.T. Yu) in Korea, based on a multigene phylogeny of GAPDH, RPB2 and Alt a1 genes. Chen et al. (2018) used the multilocus phylogenetic analyses of ITS, GAPDH and β-tubulin genes/region to characterise *A.alternata*, a causal agent of black spots of tea plant (*Camelliasinensis* (L.) Kuntze), in the Chongqing city of China. Kgatle et al. (2018) recently showed that the multi-locus phylogeny of Alt a1, RPB2, GAPDH, TEF1 and ITS genes/region successfully identified *A.alternata* causing leaf blight on sunflower (*Helianthusannuus* L.) in South Africa. Lawrence et al. (2015) provided a comprehensive taxonomic treatment of *Alternaria* with multi-locus phylogeny and accepted 27 sections in *Alternaria*, but later revised it to 28 accepted sections (Ghafri et al. 2019; Gannibal et al. 2022; Li et al. 2023). Recently, Ghafri et al. (2019) and Gannibal et al. (2022) introduced two new sections (i.e. sects. *Helianthiinficiens* and *Omanenses*) of *Alternaria* and thus, 29 sections were accepted at present (Ghafri et al. 2019; Gannibal et al. 2022; Li et al. 2023).

Chinese fir (*Cunninghamialanceolata* (Lamb.) Hook.) is an important fast-growing timber species in China and its afforestation area and timber volume rank first amongst forest plantations; it plays an important role in forest carbon sequestration, increasing farmers’ income and rural revitalisation (Yan 2020). Average timber volume is estimated at 500–800 m^3^/ha and in China, Chinese fir contributes 40% of the total commercial timber production (Zheng et al. 2016). However, Chinese fir is often damaged by many diseases and insects (Lan et al. 2015). Previous studies reported that *Alternaria* sp., *Bartaliniacunninghamiicola* Tak. Kobay. & J.Z. Zhao, *Bipolarisoryzae* (Breda de Haan) Shoemaker, *Bi. Setariae* Shoemaker, *Colletotrichumcangyuanense* Z.F. Yu, *C.fructicola* Prihast., L. Cai & K.D. Hyde, *C.gloeosporioides* (Penz.) Penz. & Sacc., *C.karsti* You L. Yang, Zuo Y. Liu, K.D. Hyde & L. Cai, *C.siamense* Prihast., L. Cai & K.D. Hyde, *Curvulariaspicifera* (Bainier) Boedijn, *Cur.muehlenbeckiae* Madrid, K.C. Cunha, Gené, Guarro & Crous, *Ceratocystiscollisensis* F.F. Liu, M.J. Wingf. & S.F. Chen, *Diaportheanhuiensis* H. Zhou & C.L. Hou, *Dia.citrichinensis* F. Huang, K.D. Hyde & Hong Y. Li, *Discosiapini* Heald, Fusariumoxysporumf.pini (R. Hartig) W.C. Snyder & H.N. Hansen, *Fusarium* sp., *Lophodermiumuncinatum* Darker, *Nigrosporasphaerica* (Sacc.) E.W. Mason and *Rhizoctoniasolani* J.G. Kühn have been identified as pathogens on Chinese fir (Anonymous 1976; Kobayashi and Zhao 1987; Wang et al. 1995; Chen 2002; Lan et al. 2015; Liu et al. 2015a; Xu and Liu 2017; Huang et al. 2018; Tian et al. 2019; Zhou and Hou 2019; Cui et al. 2020a, b; He et al. 2022). However, there is a lack of comprehensive study on *Alternaria* causing leaf blight disease on Chinese fir including diversity, occurrence and pathogenicity of the pathogens.

Surveys of fungal diseases on foliage of Chinese fir in its main cultivation regions in China were conducted from 2016 to 2020, 48 isolates of *Alternaria* spp. were collected and examined. The main aims of the present study were to determine the *Alternaria* spp. associated with leaf blight disease on Chinese fir using a polyphasic approach of fungal morphology and phylogenetic analyses, based on multi-locus sequences of ITS, SSU, LSU, GAPDH, RPB2, TEF1, Alt a1, endoPG and OPA10-2.

## ﻿Materials and methods

### ﻿Isolation of the potential fungal pathogen

A total of 48 isolates of *Alternaria* spp. were isolated from leaf blight samples of Chinese fir, which were collected in five provinces (Fujian, Henan, Hunan, Jiangsu and Shandong) in China (Suppl. material: table S1). Small pieces (2 × 3 mm) were cut from the margins of infected tissues and surface sterilised in 75% alcohol for 30 s, then in 1% sodium hypochlorite (NaOCl) for 90 s, followed by three rinses with sterile water (Huang et al. 2016), then blotted dry with sterilised filter paper, placed on 2% potato dextrose agar (PDA) Petri plates with 100 mg/l ampicillin and then cultured for 3 days at 25 °C in the dark. Fungal isolates were purified with the monosporic isolation method described by Li et al. (2007). Single-spore isolates were maintained on PDA plates. The obtained isolates were stored in the Forest Pathology Laboratory of Nanjing Forestry University. Holotype specimens of new species from this study were deposited at the China Forestry Culture Collection Center (**CFCC**), Chinese Academy of Forestry, Beijing, China.

### ﻿DNA extraction, PCR amplification and sequencing

Genomic DNA of 48 isolates was extracted using a modified CTAB method (Damm et al. 2008). The fungal plugs of each isolate were grown on the PDA plates for 5 days and then collected in a 2 ml tube. Then, 500 µl of chloroform and 500 µl of hexadecyltrimethyl ammonium bromide (CTAB) extraction buffer (0.2 M Tris, 1.4 M NaCl, 20 mM EDTA, 0.2 g/l CTAB) were added into the tubes, which were placed in a shaker at 25 °C at 200 rpm for 2 h. The mixture was centrifuged at 15,800 × *g* for 5 min. Three hundred µL of the supernatant was transferred into a new tube and 600 µl of 100% ethanol was added. The suspension was centrifuged at 15,800 × *g* for 5 min. Then, 600 µl of 70% ethanol was added into the precipitate. The suspension was centrifuged at 15,800 × *g* for 5 min and the supernatant was discarded. The DNA pellet was dried and resuspended in 30 µl ddH_2_O.

Whole or partial region/genes of nine loci were amplified. ITS and SSU were amplified with primers ITS1/ITS4 and NS1/NS4 (White et al. 1990), LSU with primers LROR/LR5 (Crous et al. 2009a), GAPDH with primers gpd1/gpd2 (Berbee et al. 1999), RPB2 with primers RPB2-5f2/fRPB2-7cr (Liu et al. 1999; Sung et al. 2007), TEF1 with primers 983F/2218R (Sung et al. 2007), Alt a1 with primers Alt-for/Alt-rev (Hong et al. 2005), endoPG and OPA10-2 with primers PG3/PG2b and OPA10-2L/OPA10-2R (Andrew et al. 2009). The information on primer pairs used are listed in Suppl. material: table S2.

The polymerase chain reaction (PCR) amplification was conducted as described by Woudenberg et al. (2015). PCR was performed in a 30 µl reaction volume containing 2 µl of genomic DNA (*ca.* 200 ng/µl), 15 µl of 2× Taq Plus Master Mix (Dye Plus) (Vazyme P212-01), 1 µl of 10 μM forward primer, 1 µl of 10 μM reverse primer and 11 µl of ddH_2_O. The PCR conditions consisted of an initial denaturation step of 4 min at 94 °C followed by 35 cycles of 30 s at 94 °C, 30 s at 55 °C and 30 s at 72 °C for ITS, GAPDH and endoPG, 35 cycles of 30 s at 94 °C, 30 s at 62 °C and 45 s at 72 °C for OPA10-2 and Alt a1, and 35 cycles of 30 s at 94 °C, 30 s at 59 °C and 60 s at 72 °C for RPB2, TEF1, LSU and SSU, and a final elongation step of 10 min at 72 °C. All DNA sequencing was performed at Shanghai Sangon Biotechnology Company (Nanjing, China). Sequences generated in this study were deposited in GenBank (Table 1).

**Table 1.** Isolates used in this study and their GenBank accession numbers.

Species name and strain number^1,2^		Locality, host / substrate	GenBank accession numbers^3^								
			SSU	LSU	ITS	GAPDH	TEF1	RPB2	Alta1	endoPG	OPA10-2
*Alternariaalternantherae* (Outgroup)	CBS 124392; HSAUP2798	China, *Solanummelongena*	KC584251	KC584506	KC584179	KC584096	KC584633	KC584374	KP123846	–	–
*A.alstroemeriae*	CBS 118808; E.G.S. 50.116^R^	USA, *Alstroemeria* sp.	KP124917	KP124447	KP124296	KP124153	KP125071	KP124764	KP123845	KP123993	KP124601
*A.alternata*	CBS 130254	India, human sputum	KP125007	KP124537	KP124383	KP124235	KP125161	KP124853	KP123931	KP124087	KP124696
	CBS 130255	India, human sputum	KP125008	KP124538	KP124384	KP124236	KP125162	KP124854	KP123932	KP124088	KP124697
	CBS 130258	India, human sputum	KP125009	KP124539	KP124385	KP124237	KP125163	KP124855	KP123933	KP124089	KP124698
*A.angustiovoidea*	CBS 195.86; E.G.S. 36.172; DAOM 185214^T^	Canada, *Euphorbiaesula*	KP124939	KP124469	KP124317	KP124173	KP125093	KP124785	JQ646398	KP124017	KP124624
*A.arborescens*	CBS 102605; E.G.S. 39.128^T^	USA, *Solanumlycopersicum*	KC584509	KC584253	AF347033	AY278810	KC584636	KC584377	AY563303	AY295028	KP124712
	CBS 124281	Denmark, *Triticum* sp.	KP125037	KP124567	KP124414	KP124265	KP125192	KP124883	KP123961	KP124118	KP124728
	CBS 124282	Denmark, *Hordeumvulgare*	KP125038	KP124568	KP124415	KP124266	KP125193	KP124884	KP123962	KP124119	KP124729
	CPC 25266	Austria, *Pyrus* sp.	KP125041	KP124571	KP124418	KP124269	KP125196	KP124887	KP123965	KP124122	KP124732
*A.astragali*	CBS 127672; E.G.S. 52.122^T^	USA, *Astragalusbisulcatus*	KP125006	KP124536	KP124382	KP124234	KP125160	KP124852	KP123930	KP124086	KP124695
*A.betae-kenyensis*	CBS 118810; E.G.S. 49.159; IMI 385709^T^	Kenya, *Betavulgaris* var. cicla	KP125042	KP124572	KP124419	KP124270	KP125197	KP124888	KP123966	KP124123	KP124733
*A.brassicinae*	CBS 118811; E.G.S. 35.158^T^	USA, *Brassicaoleracea*	KP124978	KP124508	KP124356	KP124210	KP125132	KP124824	KP123904	KP124057	KP124667
*A.broussonetiae*	CBS 121455; E.G.S. 50.078^T^	China, *Broussonetiapapyrifera*	KP124992	KP124522	KP124368	KP124220	KP125146	KP124838	KP123916	KP124072	KP124681
*A.burnsii*	CBS 108.27	Unknown, *Gomphrenaglobosa*	KC584601	KC584343	KC584236	KC584162	KC584727	KC584468	KP123850	KP123997	KP124605
	CBS 107.38; E.G.S. 06.185^T^	India, *Cuminumcyminum*	KP125043	KP124573	KP124420	JQ646305	KP125198	KP124889	KP123967	KP124124	KP124734
	CBS 130264	India, human sputum	KP125048	KP124578	KP124425	KP124275	KP125203	KP124894	KP123972	KP124129	KP124739
*A.caudata*	CBS 121544; E.G.S. 38.022^R^	USA, *Cucumissativus*	KP124995	KP124525	KP124371	KP124223	KP125149	KP124841	KP123919	KP124075	KP124684
*A.citri*	CBS 107.27; ATCC 24463; QM 1736^ET^	USA, *Citruslimonium*	KP124921	KP124451	KP124300	KP124157	KP125075	KP124768	KP123849	KP123996	KP124604
*A.cinerariae*	CBS 612.72; DSM 62012^ET^	Germany, *Seneciocineraria*	KP124930	KP124460	KP124308	KP124165	KP125084	KP124777	KP123861	KP124008	KP124615
*A.citrimacularis*	CBS 102596; E.G.S. 45.090^T^	USA, *Citrusjambhiri*	KP124950	KP124480	KP124328	KP124183	KP125104	KP124796	KP123877	KP124030	KP124637
*A.citriarbusti*	CBS 102598; E.G.S. 46.141^T^	USA, *Minneolatangelo*	KP124951	KP124481	KP124329	KP124184	KP125105	KP124797	KP123878	KP124031	KP124638
*A.citricancri*	CBS 119543; E.G.S. 12.160^T^	USA, *Citrusparadisi*	KP124985	KP124515	KP124363	KP124215	KP125139	KP124831	KP123911	KP124065	KP124674
***A.cunninghamiicola***	**DSQ3-2**	**China, *Cunninghamialanceolata* leaf**	**OR229504**	**OR229647**	**OR229442**	**OR252424**	**OR233910**	**OR252520**	**OR252376**	**OR252472**	**OR233862**
	**DSQ3-2-1**	**China, *Cu.lanceolata* leaf**	**OR229505**	**OR229648**	**OR229443**	**OR252425**	**OR233911**	**OR252521**	**OR252377**	**OR252473**	**OR233863**
	**DSQ3-2-2**	**China, *Cu.lanceolata* leaf**	**OR229506**	**OR229649**	**OR229444**	**OR252426**	**OR233912**	**OR252522**	**OR252378**	**OR252474**	**OR233864**
	**DSQ3-2-3**	**China, *Cu.lanceolata* leaf**	**OR229507**	**OR229650**	**OR229445**	**OR252427**	**OR233913**	**OR252523**	**OR252379**	**OR252475**	**OR233865**
	**DSQ3-2-4**	**China, *Cu.lanceolata* leaf**	**OR229508**	**OR229651**	**OR229446**	**OR252428**	**OR233914**	**OR252524**	**OR252380**	**OR252476**	**OR233866**
*A.daucifolii*	CBS 118812; E.G.S. 37.050^T^	USA, *Daucuscarota*	KC584525	KC584269	KC584193	KC584112	KC584652	KC584393	KP123905	KP124058	KP124668
*A.destruens*	CBS 121454; E.G.S. 46.069^T^	USA, *Cuscutagronovii*	KP124991	KP124521	–	AY278812	KP125145	KP124837	JQ646402	KP124071	KP124680
***A.dongshanqiaoensis***	**DSQ2-2**	**China, *Cu.lanceolata* leaf**	**OR229495**	**OR229638**	**OR229433**	**OR252415**	**OR233901**	**OR252511**	**OR252367**	**OR252463**	**OR233853**
	**DSQ2-2-1**	**China, *Cu.lanceolata* leaf**	**OR229496**	**OR229639**	**OR229434**	**OR252416**	**OR233902**	**OR252512**	**OR252368**	**OR252464**	**OR233854**
	**DSQ2-2-2**	**China, *Cu.lanceolata* leaf**	**OR229497**	**OR229640**	**OR229435**	**OR252417**	**OR233903**	**OR252513**	**OR252369**	**OR252465**	**OR233855**
	**DSQ2-2-3**	**China, *Cu.lanceolata* leaf**	**OR229498**	**OR229641**	**OR229436**	**OR252418**	**OR233904**	**OR252514**	**OR252370**	**OR252466**	**OR233856**
	**HN43-6-1**	**China, *Cu.lanceolata* leaf**	**OR229499**	**OR229642**	**OR229437**	**OR252419**	**OR233905**	**OR252515**	**OR252371**	**OR252467**	**OR233857**
	**HN43-6-1-1**	**China, *Cu.lanceolata* leaf**	**OR229500**	**OR229643**	**OR229438**	**OR252420**	**OR233906**	**OR252516**	**OR252372**	**OR252468**	**OR233858**
	**HN43-6-1-2**	**China, *Cu.lanceolata* leaf**	**OR229501**	**OR229644**	**OR229439**	**OR252421**	**OR233907**	**OR252517**	**OR252373**	**OR252469**	**OR233859**
	**HN43-6-1-3**	**China, *Cu.lanceolata* leaf**	**OR229502**	**OR229645**	**OR229440**	**OR252422**	**OR233908**	**OR252518**	**OR252374**	**OR252470**	**OR233860**
	**HN43-6-1-4**	**China, *Cu.lanceolata* leaf**	**OR229503**	**OR229646**	**OR229441**	**OR252423**	**OR233909**	**OR252519**	**OR252375**	**OR252471**	**OR233861**
*A.dumosa*	CBS 102604; E.G.S. 45.007^T^	Israel, *Minneolatangelo*	KP124956	KP124486	KP124334	AY562410	KP125110	KP124802	AY563305	KP124035	KP124643
*A.eichhorniae*	CBS 489.92; ATCC 22255; ATCC 46777; IMI 121518^T^	India, *Eichhorniacrassipes*	KP125049	KP124579	KC146356	KP124276	KP125204	KP124895	KP123973	KP124130	KP124740
*A.gaisen*	CBS 632.93; E.G.S. 90.512^R^	Japan, *Pyruspyrifolia*	KC584531	KC584275	KC584197	KC584116	KC584658	KC584399	KP123974	AY295033	KP124742
	CBS 118488; E.G.S. 90.391^R^	Japan, *Pyruspyrifolia*	KP125051	KP124581	KP124427	KP124278	KP125206	KP124897	KP123975	KP124132	KP124743
	CPC 25268	Portugal, unknown	KP125052	KP124582	KP124428	KP124279	KP125207	KP124898	KP123976	KP124133	KP124744
*A.godetiae*	CBS 117.44; E.G.S. 06.190; VKM F-1870^T^	Denmark, *Godetia* sp.	KP124925	KP124455	KP124303	KP124160	KP125079	KP124772	KP123854	KP124001	KP124609
*A.gossypina*	CBS 104.32^T^	Zimbabwe, *Gossypium* sp.	KP125054	KP124584	KP124430	JQ646312	KP125209	KP124900	JQ646395	KP124135	KP124746
*A.grisea*	CBS 107.36^T^	Indonesia, soil	KP125055	KP124585	KP124431	JQ646310	KP125210	KP124901	JQ646393	KP124136	KP124747
*A.herbiphorbicola*	CBS 119408; E.G.S. 40.140^T^	USA, *Euphorbiaesula*	KP124984	KP124514	KP124362	JQ646326	KP125138	KP124830	JQ646410	KP124064	KP124673
***A.hunanensis***	**HN43-10-2**	**China, *Cu.lanceolata* leaf**	**OR229486**	**OR229629**	**OR229424**	**OR252406**	**OR233892**	**OR252502**	**OR252358**	**OR252454**	**OR233844**
	**HN43-10-2-1**	**China, *Cu.lanceolata* leaf**	**OR229487**	**OR229630**	**OR229425**	**OR252407**	**OR233893**	**OR252503**	**OR252359**	**OR252455**	**OR233845**
	**HN43-10-2-2**	**China, *Cu.lanceolata* leaf**	**OR229488**	**OR229631**	**OR229426**	**OR252408**	**OR233894**	**OR252504**	**OR252360**	**OR252456**	**OR233846**
	**HN43-10-2-3**	**China, *Cu.lanceolata* leaf**	**OR229489**	**OR229632**	**OR229427**	**OR252409**	**OR233895**	**OR252505**	**OR252361**	**OR252457**	**OR233847**
	**HN43-10-2-4**	**China, *Cu.lanceolata* leaf**	**OR229490**	**OR229633**	**OR229428**	**OR252410**	**OR233896**	**OR252506**	**OR252362**	**OR252458**	**OR233848**
*A.interrupta*	CBS 102603; E.G.S. 45.011^T^	Israel, *Minneolatangelo*	KP124955	KP124485	KP124333	KP124188	KP125109	KP124801	KP123882	KP124034	KP124642
*A.iridiaustralis*	CBS 118486; E.G.S. 43.014^T^	Australia, *Iris* sp.	KP125059	KP124589	KP124435	KP124284	KP125214	KP124905	KP123981	KP124140	KP124751
	CBS 118487; E.G.S. 44.147^R^	Australia, *Iris* sp.	KP125060	KP124590	KP124436	KP124285	KP125215	KP124906	KP123982	KP124141	KP124752
*A.jacinthicola*	CBS 133751; MUCL 53159^T^	Mali, *Eichhorniacrassipes*	KP125062	KP124592	KP124438	KP124287	KP125217	KP124908	KP123984	KP124143	KP124754
	CPC 25267	Unknown, Cucumismelovar.inodorus	KP125063	KP124593	KP124439	KP124288	KP125218	KP124909	KP123985	KP124144	KP124755
*A.kikuchiana*	CBS 107.53; DSM 3187; IFO 5778^HT^	Japan, *Pyruspyrifolia*	KP124927	KP124457	KP124305	KP124162	KP125081	KP124774	KP123858	KP124005	KP124613
*A.koreana*	SPL2-1	Korea, *Atractylodesovata*	–	–	LC621613	LC621647	LC621715	LC621681	LC631831	LC631844	LC631857
*A.koreana*	SPL2-4	Korea, *Atractylodesovata*	–	–	LC621615	LC621649	LC621717	LC621683	LC631832	LC631845	LC631858
***A.kunyuensis***	**XXG21**	**China, *Cu.lanceolata* leaf**	**OR229515**	**OR229658**	**OR229453**	**OR252435**	**OR233921**	**OR252531**	**OR252387**	**OR252483**	**OR233873**
	**XXG22**	**China, *Cu.lanceolata* leaf**	**OR229516**	**OR229659**	**OR229454**	**OR252436**	**OR233922**	**OR252532**	**OR252388**	**OR252484**	**OR233874**
	**XXG26-2**	**China, *Cu.lanceolata* leaf**	**OR229517**	**OR229660**	**OR229455**	**OR252437**	**OR233923**	**OR252533**	**OR252389**	**OR252485**	**OR233875**
	**XXG31**	**China, *Cu.lanceolata* leaf**	**OR229518**	**OR229661**	**OR229456**	**OR252438**	**OR233924**	**OR252534**	**OR252390**	**OR252486**	**OR233876**
	**XXG30**	**China, *Cu.lanceolata* leaf**	**OR229519**	**OR229662**	**OR229457**	**OR252439**	**OR233925**	**OR252535**	**OR252391**	**OR252487**	**OR233877**
	**XXG12-2**	**China, *Cu.lanceolata* leaf**	**OR229520**	**OR229663**	**OR229458**	**OR252440**	**OR233926**	**OR252536**	**OR252392**	**OR252488**	**OR233878**
*A.lini*	CBS 106.34; E.G.S. 06.198; DSM 62019; MUCL 10030^T^	Unknown, *Linumusitatissimum*	KP124924	KP124454	Y17071	JQ646308	KP125078	KP124771	KP123853	KP124000	KP124608
*A.limoniasperae*	CBS 102595; E.G.S. 45.100^T^	USA, *Citrusjambhiri*	KC584540	KC584284	FJ266476	AY562411	KC584666	KC584408	AY563306	KP124029	KP124636
*A.longipes*	CBS 113.35	Unknown, *Nicotianatabacum*	KP125064	KP124594	KP124440	KP124289	KP125219	KP124910	KP123986	KP124145	KP124756
	CBS 917.96	USA, *Nicotianatabacum*	KP125066	KP124596	KP124442	KP124291	–	KP124912	KP123988	KP124148	KP124759
***A.longqiaoensis***	**HN43-14**	**China, *Cu.lanceolata* leaf**	**OR229491**	**OR229634**	**OR229429**	**OR252411**	**OR233897**	**OR252507**	**OR252363**	**OR252459**	**OR233849**
	**HN43-14-1**	**China, *Cu.lanceolata* leaf**	**OR229492**	**OR229635**	**OR229430**	**OR252412**	**OR233898**	**OR252508**	**OR252364**	**OR252460**	**OR233850**
	**HN43-14-2**	**China, *Cu.lanceolata* leaf**	**OR229493**	**OR229636**	**OR229431**	**OR252413**	**OR233899**	**OR252509**	**OR252365**	**OR252461**	**OR233851**
	**HN43-14-3**	**China, *Cu.lanceolata* leaf**	**OR229494**	**OR229637**	**OR229432**	**OR252414**	**OR233900**	**OR252510**	**OR252366**	**OR252462**	**OR233852**
*A.mali*	CBS 106.24; E.G.S. 38.029; ATCC 13963^T^	USA, *Malussylvestris*	KP124919	KP124449	KP124298	KP124155	KP125073	KP124766	KP123847	AY295020	JQ800620
*A.malvae*	CBS 447.86	Marocco, *Malva* sp.	KP124940	KP124470	KP124318	JQ646314	KP125094	KP124786	JQ646397	KP124018	KP124625
*A.palandui*	CBS 121336; E.G.S. 37.005; ATCC 11680^T^	USA, *Allium* sp.	KP124987	KP124517	KJ862254	KJ862255	KP125141	KP124833	KJ862259	KP124067	KP124676
*A.pellucida*	CBS 479.90; E.G.S. 29.028^T^	Japan, *Citrusunshiu*	KP124941	KP124471	KP124319	KP124174	KP125095	KP124787	KP123870	KP124019	KP124626
*A.perangusta*	CBS 102602; E.G.S. 44.160^T^	Turkey, *Minneolatangelo*	KP124954	KP124484	KP124332	KP124187	KP125108	KP124800	KP123881	AY295023	KP124641
*A.platycodonis*	CBS 121348; E.G.S. 50.070^T^	China, *Platycodongrandiflflorus*	KP124990	KP124520	KP124367	KP124219	KP125144	KP124836	KP123915	KP124070	KP124679
*A.postmessia*	CBS 119399; E.G.S. 39.189^T^	USA, *Minneolatangelo*	KP124983	KP124513	KP124361	JQ646328	KP125137	KP124829	KP123910	KP124063	KP124672
*A.pulvinifungicola*	CBS 194.86; E.G.S. 04.090; QM 1347^T^	USA, *Quercus* sp.	KP124938	KP124468	KP124316	KP124172	KP125092	KP124784	KP123869	KP124016	KP124623
*A.rhadina*	CBS 595.93^T^	Japan, *Pyruspyrifolia*	KP124942	KP124472	KP124320	KP124175	KP125096	KP124788	JQ646399	KP124020	KP124627
*A.sanguisorbae*	CBS 121456; E.G.S. 50.080; HSAUP 9600197^T^	China, *Sanguisorbaoffificinalis*	KP124993	KP124523	KP124369	KP124221	KP125147	KP124839	KP123917	KP124073	KP124682
*A.seleniiphila*	CBS 127671; E.G.S. 52.121^T^	USA, *Stanleyapinnata*	KP125005	KP124535	KP124381	KP124233	KP125159	KP124851	KP123929	KP124085	KP124694
*A.septorioides*	CBS 175.80	Italy, unknown	KP124935	KP124465	KP124313	JQ646324	KP125089	KP124781	KP123866	KP124013	KP124620
***A.shandongensis***	**SDHG12**	**China, *Cu.lanceolata* leaf**	**OR229509**	**OR229652**	**OR229447**	**OR252429**	**OR233915**	**OR252525**	**OR252381**	**OR252477**	**OR233867**
	**SDHG12-1**	**China, *Cu.lanceolata* leaf**	**OR229510**	**OR229653**	**OR229448**	**OR252430**	**OR233916**	**OR252526**	**OR252382**	**OR252478**	**OR233868**
	**SDHG12-2**	**China, *Cu.lanceolata* leaf**	**OR229511**	**OR229654**	**OR229449**	**OR252431**	**OR233917**	**OR252527**	**OR252383**	**OR252479**	**OR233869**
***A.shandongensis***	**SDHG12-3**	**China, *Cu.lanceolata* leaf**	**OR229512**	**OR229655**	**OR229450**	**OR252432**	**OR233918**	**OR252528**	**OR252384**	**OR252480**	**OR233870**
	**SDHG12-4**	**China, *Cu.lanceolata* leaf**	**OR229513**	**OR229656**	**OR229451**	**OR252433**	**OR233919**	**OR252529**	**OR252385**	**OR252481**	**OR233871**
	**LY15**	**China, *Cu.lanceolata* leaf**	**OR229514**	**OR229657**	**OR229452**	**OR252434**	**OR233920**	**OR252530**	**OR252386**	**OR252482**	**OR233872**
*A.soliaegyptiaca*	CBS 103.33; E.G.S. 35.182; IHEM 3319^T^	Egypt, soil	KP124923	KP124453	KP124302	KP124159	KP125077	KP124770	KP123852	KP123999	KP124607
*A.tenuis*	CBS 126910	USA, soil	KP125003	KP124533	KP124379	KP124231	KP125157	KP124849	KP123927	KP124083	KP124692
*A.tenuissima*	CBS 620.83; ATCC 15052^ET^	USA, *Nicotianatabacum*	KP124937	KP124467	KP124315	KP124171	KP125091	KP124783	KP123868	KP124015	KP124622
*A.tomato*	CBS 103.30	Unknown, *Solanumlycopersicum*	KP125069	KP124599	KP124445	KP124294	KP125224	KP124915	KP123991	KP124151	KP124762
	CBS 114.35	Unknown, *Solanumlycopersicum*	KP125070	KP124600	KP124446	KP124295	KP125225	KP124916	KP123992	KP124152	KP124763
*A.tomaticola*	CBS 118814; E.G.S. 44.048^T^	USA, *Solanumlycopersicum*	KP124979	KP124509	KP124357	KP124211	KP125133	KP124825	KP123906	KP124059	KP124669
*A.toxicogenica*	CBS 102600; E.G.S. 39.181; ATCC 38963^T^	USA, *Citrusreticulata*	KP124953	KP124483	KP124331	KP124186	KP125107	KP124799	KP123880	KP124033	KP124640
*A.turkisafria*	CBS 102599; E.G.S. 44.166^T^	Turkey, *Minneolatangelo*	KP124952	KP124482	KP124330	KP124185	KP125106	KP124798	KP123879	KP124032	KP124639
*A.vaccinii*	CBS 118818; E.G.S. 31.032^T^	USA, *Vaccinium* sp.	KP124981	KP124511	KP124359	KP124213	KP125135	KP124827	KP123908	KP124061	KP124671
***A.xinyangensis***	**ZLS1**	**China, *Cu.lanceolata* leaf**	**OR229521**	**OR229664**	**OR229459**	**OR252441**	**OR233927**	**OR252537**	**OR252393**	**OR252489**	**OR233879**
	**ZLS1-1**	**China, *Cu.lanceolata* leaf**	**OR229522**	**OR229665**	**OR229460**	**OR252442**	**OR233928**	**OR252538**	**OR252394**	**OR252490**	**OR233880**
	**ZLS1-2**	**China, *Cu.lanceolata* leaf**	**OR229523**	**OR229666**	**OR229461**	**OR252443**	**OR233929**	**OR252539**	**OR252395**	**OR252491**	**OR233881**
	**ZLS1-3**	**China, *Cu.lanceolata* leaf**	**OR229524**	**OR229667**	**OR229462**	**OR252444**	**OR233930**	**OR252540**	**OR252396**	**OR252492**	**OR233882**
	**ZLS1-4**	**China, *Cu.lanceolata* leaf**	**OR229525**	**OR229668**	**OR229463**	**OR252445**	**OR233931**	**OR252541**	**OR252397**	**OR252493**	**OR233883**
	**XYXY06**	**China, *Cu.lanceolata* leaf**	**OR229526**	**OR229669**	**OR229464**	**OR252446**	**OR233932**	**OR252542**	**OR252398**	**OR252494**	**OR233884**
	**XYXY8-2**	**China, *Cu.lanceolata* leaf**	**OR229527**	**OR229670**	**OR229465**	**OR252447**	**OR233933**	**OR252543**	**OR252399**	**OR252495**	**OR233885**
	**XYXY16**	**China, *Cu.lanceolata* leaf**	**OR229528**	**OR229671**	**OR229466**	**OR252448**	**OR233934**	**OR252544**	**OR252400**	**OR252496**	**OR233886**
	**XYXY15**	**China, *Cu.lanceolata* leaf**	**OR229529**	**OR229672**	**OR229467**	**OR252449**	**OR233935**	**OR252545**	**OR252401**	**OR252497**	**OR233887**
	**XYXY15-1**	**China, *Cu.lanceolata* leaf**	**OR229530**	**OR229673**	**OR229468**	**OR252450**	**OR233936**	**OR252546**	**OR252402**	**OR252498**	**OR233888**
	**XYXY15-2**	**China, *Cu.lanceolata* leaf**	**OR229531**	**OR229674**	**OR229469**	**OR252451**	**OR233937**	**OR252547**	**OR252403**	**OR252499**	**OR233889**
	**XYXY15-3**	**China, *Cu.lanceolata* leaf**	**OR229532**	**OR229675**	**OR229470**	**OR252452**	**OR233938**	**OR252548**	**OR252404**	**OR252500**	**OR233890**
	**XYXY15-4**	**China, *Cu.lanceolata* leaf**	**OR229533**	**OR229676**	**OR229471**	**OR252453**	**OR233939**	**OR252549**	**OR252405**	**OR252501**	**OR233891**
*A.yali-inficiens*	CBS 121547; E.G.S. 50.048^T^	China, *Pyrusbretschneideri*	KP124996	KP124526	KP124372	KP124224	KP125150	KP124842	KP123920	KP124076	KP124685

1 ATCC: American Type Culture Collection, Manassas, VA, USA; CBS: Culture collection of the Westerdijk Fungal Biodiversity Institute, Utrecht, The Netherlands; CPC: Personal collection of P.W. Crous, Utrecht, The Netherlands; DAOM: Canadian Collection of Fungal Cultures, Ottawa, Canada; DSM: German Collection of Microorganisms and Cell Cultures, Leibniz Institute, Braunschweig, Germany; E.G.S.: Personal collection of Dr. E.G. Simmons; HKUCC: The University of Hong Kong Culture Collection, Hong Kong, China; HSAUP: Department of Plant Pathology, Shandong Agricultural University, China; IFO: Institute for Fermentation Culture Collection, Osaka, Japan; IHEM: Biomedical Fungi and Yeast Collection of the Belgian Co-ordinated Collections of Micro-organisms (BCCM), Brussels, Belgium; IMI: Culture collection of CABI Europe UK Centre, Egham UK; LCP: Laboratory of Cryptogamy, National Museum of Natural History, Paris, France; MAFF: MAFF Genebank Project, Ministry of Agriculture, Forestry and Fisherie, Tsukuba, Japan; MUCL: (Agro)Industrial Fungi and Yeast Collection of the Belgian Co-ordinated Collections of Micro-organisms (BCCM), Louvain-la-Neuve, Belgium; QM: Quarter Master Culture Collection, Amherst, MA, USA; VKM: All-Russian Collection of Microorganisms, Moscow, Russia. T: ex-type isolate; ET: ex-epitype isolate; HT: ex-holotype isolate; R: representative isolate. 3 Bold accession numbers are generated in other studies; np: no product.

### ﻿Phylogenetic analyses

The sequences generated in this study were compared against nucleotide sequences in GenBank using BLAST to determine closely-related taxa. Alignments of different loci, including the sequences obtained from this study and the ones downloaded from GenBank, were initially performed with the MAFFT v.7 online server (https://mafft.cbrc.jp/alignment/server/) (Katoh and Standley 2013) and then manually adjusted in MEGA v. 10 (Kumar et al. 2018). The post-alignment sequences of multiple loci were concatenated in PhyloSuite software (Zhang et al. 2020). Maximum-Likelihood (ML) and Bayesian Inference (BI) were run in PhyloSuite software using IQ-TREE ver. 1.6.8 (Nguyen et al. 2015) and MrBayes v. 3.2.6 (Ronquist et al. 2012), respectively. ModelFinder was used to carry out statistical selection of best-fit models of nucleotide substitution using the corrected Akaike information criterion (AIC) (Kalyaanamoorthy et al. 2017). For ML analyses, the default parameters were used, and bootstrap support (BS) was carried out using the rapid bootstrapping algorithm with the automatic halt option. Bayesian analyses included two parallel runs of 2,000,000 generations, with the stop rule option and a sampling frequency set to each 1,000 generations. The 50% majority rule consensus trees and posterior probability (PP) values were calculated after discarding the first 25% of the samples as burn-in. Phylogenetic trees were visualised in FigTree v. 1.4.2 (http://tree.bio.ed.ac.uk/software/figtree/) (Rambaut 2014).

Phylogenetically-related, but ambiguous species were analysed using the genealogical concordance phylogenetic species recognition (GCPSR) model by performing a pairwise homoplasy index (PHI) test as described by Quaedvlieg et al. (2014). The PHI test was performed in SplitsTree4 (Huson 1998; Huson and Bryant 2006) in order to determine the recombination level within phylogenetically closely-related species using a concatenated multi-locus dataset (ITS, SSU, LSU, GAPDH, RPB2, TEF1, Alt a1, endoPG and OPA10-2). If the pairwise-homoplasy index results were below a 0.05 threshold (Ф_w_ < 0.05), it indicates significant recombination present in the dataset. The relationship amongst the closely-related species was visualised by constructing splits graphs.

### ﻿Morphological study

One representative isolate was randomly selected from each *Alternaria* species for morphological research according to the method of Simmons (2007). Mycelial plugs (5 mm) of purified cultures were transferred from the growing edge of 5-d-old cultures to the centre of 7-mm-diameter potato carrot agar (PCA) plates (Crous et al. 2009b) in triplicate at 25 °C. Colony diameters were measured from 3 to 6 days to calculate mycelial growth rates (mm/d). Colony colour, size and density were also recorded. The morphology and size of conidial chains were studied and recorded using a Zeiss stereo microscope (SteRo Discovery v.20). The shape, colour and size of conidiophores and conidia were observed using a ZEISS Axio Imager A2m microscope (ZEISS, Germany) with differential interference contrast (DIC) optics. At least 30 measurements per structure were performed using Carl Zeiss Axio Vision software to determine their sizes, unless no or fewer individual structures were produced.

### ﻿Pathogenicity tests

Seven representative isolates (ZLS1, DSQ2-2, SDHG12, XXG21, HN43-10-2, HN43-14 and DSQ3-2) of *Alternaria* species were selected for the pathogenicity test on detached leaves of Chinese fir collected from 1-year-old Chinese fir plants on the campus of Nanjing Forestry University, Jiangsu, China.

For in-vitro inoculation, detached leaves were surface-sterilised with 75% ethanol, washed three times with sterile water and air-dried on sterile filter paper. A 10 µl aliquot of conidial suspension (1.0 × 10^6^ conidia/ml) was transferred to a sterile plastic tube (20 × 6 mm), in which a leaf was placed so that the base of the leaf was immersed in the conidial suspension. The control was treated with the same amount of double-distilled water. Leaves in the tubes were then placed in plastic trays (40 × 25 cm), covered with a piece of plastic wrap to maintain relative humidity at 99% and incubated at 25 °C in the dark for 5 days. Each treatment had twelves replicates and the experiment was conducted three times. Symptom development on each detached leaf was evaluated by determining the means of lesion lengths at 5 days post-inoculation (dpi). The data were analysed by analysis of variance (ANOVA) using SPSS v. 18 software. LSD’s range test was used to determine significant differences amongst or between different treatments. Origin v. 8.0 software was used to draw histograms (Li et al. 2020). Pathogens were re-isolated from the resulting lesions and identified as described above.

## ﻿Results

### ﻿Phylogenetic analyses

**Figure 1.**
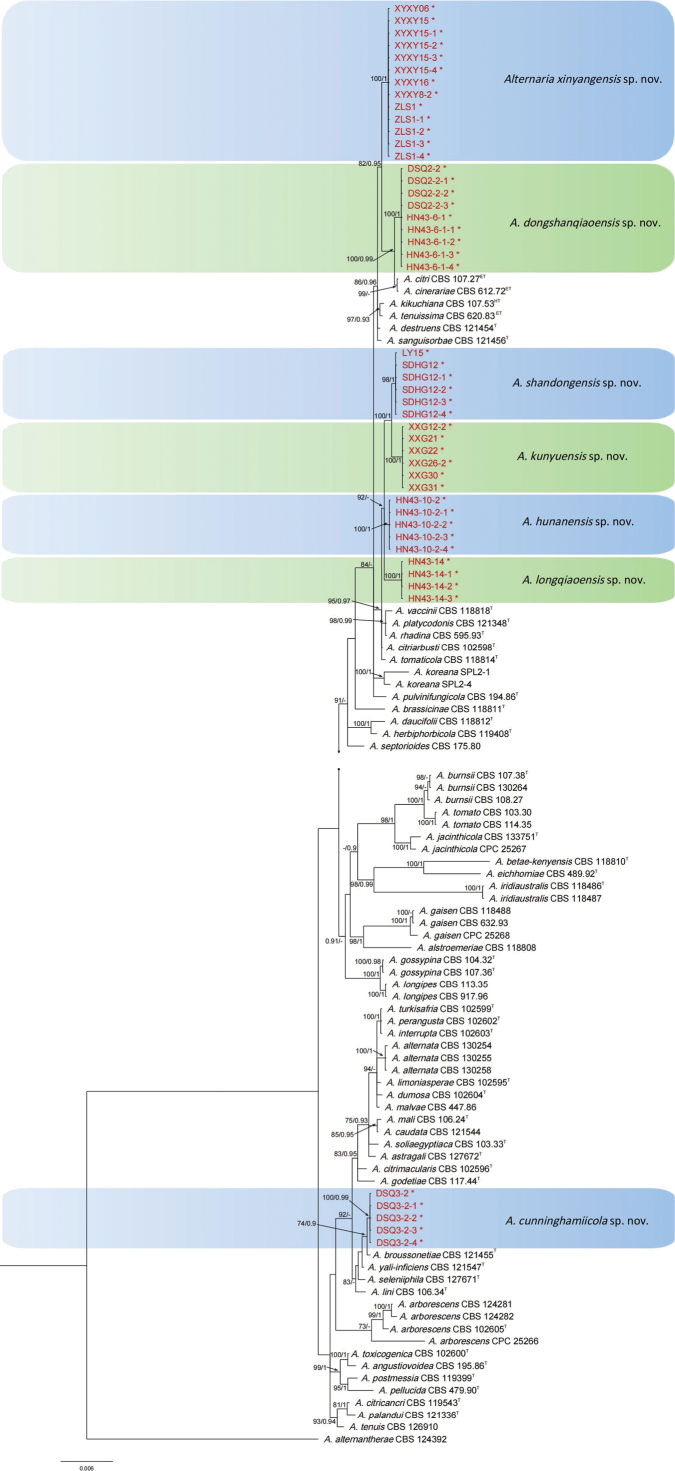
Phylogenetic relationships of 116 isolates of the *Alternaria* species complex with related taxa with concatenated sequences of the SSU, LSU, ITS, GAPDH, RPB2, TEF1, Alt a1, endoPG and OPA10-2 loci using Bayesian inference (BI) and Maximum-likelihood (ML) methods. Bootstrap support values from ML ≥ 70% and BI posterior values ≥ 0.9 are shown at nodes (ML/BI). *Alternariaalternantherae*CBS 124392 was the outgroup. * and red font indicates strains of this study. ^T^ indicates the ex-type strains, ^ET^ indicates the ex-epitype strains, ^HT^ indicates the ex-holotype strains.

**Figure 2.**
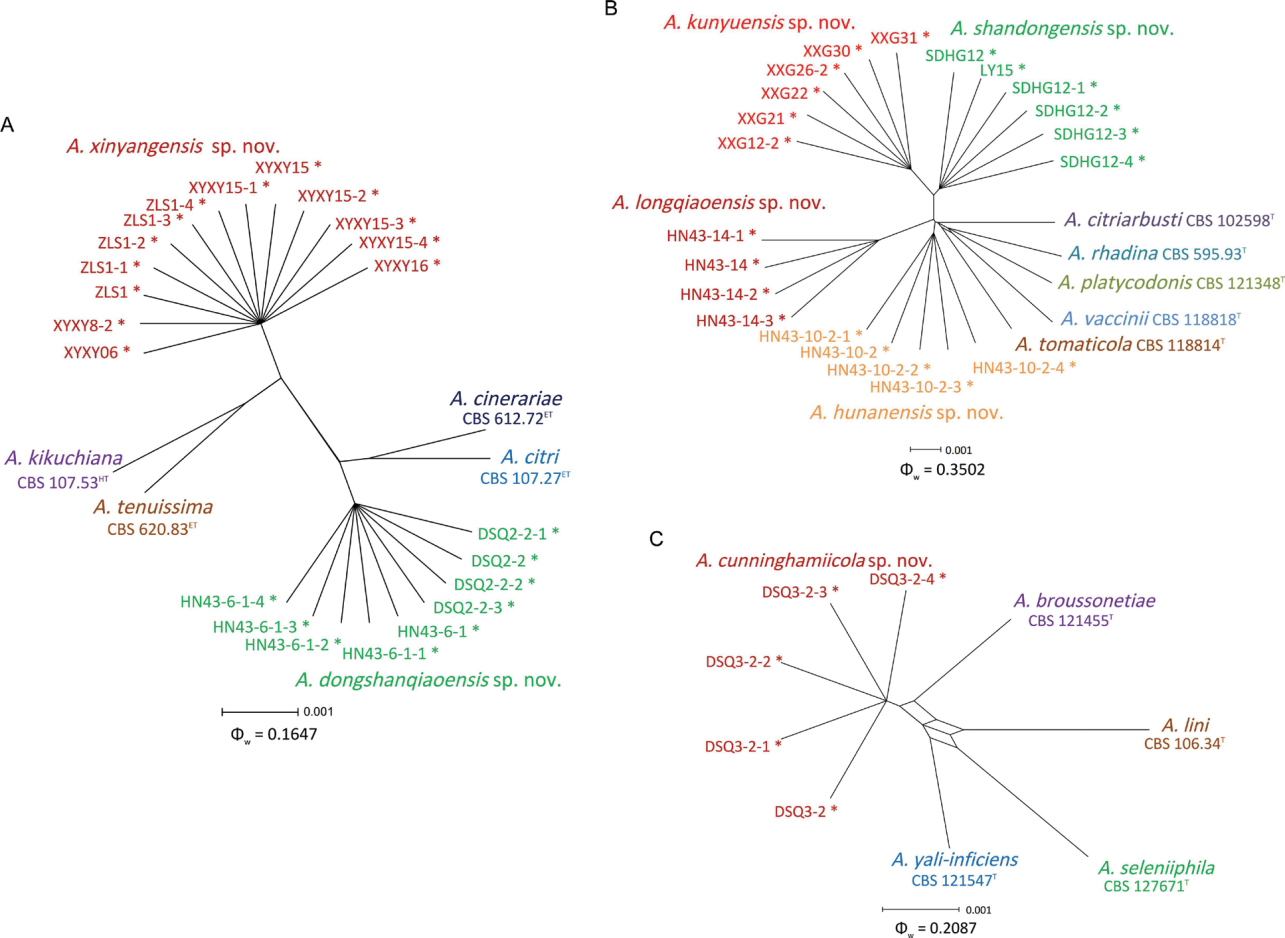
Splitgraphs showing the results of the pairwise homoplasy index (PHI) test of newly described taxa and closely-related species using both LogDet transformation and splits decomposition **A** the PHI of *Alternariaxinyangensis* sp. nov. and *A.dongshanqiaoensis* sp. nov. with their phylogenetically related isolates or species **B** the PHI of *A.shandongensis* sp. nov., *A.kunyuensis* sp. nov., *A.hunanensis* sp. nov. and *A.longqiaoensis* sp. nov. with their phylogenetically related isolates or species **C** the PHI of *A.cunninghamiicola* sp. nov. with their phylogenetically-related isolates or species. PHI test value (Φ_w_) < 0.05 indicate significant recombination within a dataset. * indicates strains of this study. ^T^ indicates the ex-type strains, ^ET^ indicates the ex-epitype strains, ^HT^ indicates the ex-holotype strains.

A total of 48 *Alternaria* isolates from Chinese fir were subjected to multi-locus phylogenetic analyses for *Alternaria* spp. with concatenated sequences of ITS, SSU, LSU, GAPDH, RPB2, TEF1, Alt a1, endoPG and OPA10-2. The data matrix contained a total of 5460 characters with gaps (Alt a1: 1–453, GAPDH: 454–952, ITS: 953–1462, LSU: 1463–2349, OPA10-2: 2350–3013, endoPG: 3014–3414, RPB2: 3415–4170, SSU: 4171–5167, TEF1: 5168–5460). *Alternariaalternantherae* Holcomb & Antonop. CBS 124392 was used as the out-group. The Maximum-likelihood (ML) and Bayesian Inference (BI) phylogenetic analyses showed that 48 isolates clustered into seven clades distantly from any known species (Fig. 1). Of these, 13 isolates clustered distantly from any known species with high support (ML-BS/BI-PP = 100/1) and closely related to *A.dongshanqiaoensis* sp. nov. (this study, DSQ2-2), *A.citri* (Penz.) Mussat (ex-epitype, CBS 107.27), *A.cinerariae* Hori & Enjoji (ex-type, CBS 612.72) and *A.kikuchiana* S. Tanaka (ex-type, CBS 107.53), are herein described as a new taxon, namely *A.xinyangensis* sp. nov. (Fig. 1). The results showed that nine isolates clustered in a distinct clade with high support (ML-BS/BI-PP = 100/1), which was distinct from all other known species and closely related to *A.xinyangensis* sp. nov. (this study, ZLS1), *A.citri* (ex-epitype, CBS 107.27), *A.cinerariae* (ex-type, CBS 612.72) and *A.kikuchiana* (ex-type, CBS 107.53), namely *A.dongshanqiaoensis* sp. nov. (Fig. 1). When applying the GCPSR concept to these isolates, the concatenated sequence dataset of nine-loci (ITS, SSU, LSU, GAPDH, RPB2, TEF1, Alt a1, endoPG and OPA10-2) was subjected to the PHI test and the result showed that no significant recombination was detected amongst these isolates/taxa (Φw = 0.1647) (Fig. 2A). It was a solid support for the proposition that these isolates belonged to six distinct taxa.

The ML/BI phylogenetic analyses also showed that *A.shandongensis* (six isolates, ML-BS/BI-PP = 98/1), *A.kunyuensis* (six isolates, ML-BS/BI-PP = 100/1), *A.hunanensis* (five isolates, ML-BS/BI-PP = 100/1) and *A.longqiaoensis* (four isolates, ML-BS/BI-PP = 100/1) clustered in four distinct clades, which were distinct from all other known species and closely related to *A.vaccinii* E.G. Simmons (ex-type, CBS 118818), *A.platycodonis* Z.Y. Zhang & H. Zhang (ex-type, CBS 121348), *A.rhadina* E.G. Simmons (ex-type, CBS 595.93), *A.citriarbusti* E.G. Simmons (ex-type, CBS 102598) and *A.tomaticola* E.G. Simmons & Chellemi (ex-type, CBS 118814) (Fig. 1). When applying the GCPSR concept to these isolates, the concatenated sequence dataset of nine-loci (ITS, SSU, LSU, GAPDH, RPB2, TEF1, Alt a1, endoPG and OPA10-2) was subjected to the PHI test and showed that no significant recombination was detected amongst these isolates/taxa (Φ_w_ = 0.3502) (Fig. 2B). It was a solid support for the proposition that these isolates belonged to nine distinct taxa.

Phylogenetic analyses also showed that the five isolates (DSQ3-2, DSQ3-2-1, DSQ3-2-2, DSQ3-2-3 and DSQ3-2-4) clustered in a distinct clade with high support (ML-BS/BI-PP = 100/0.99), which was distinct from all other known species and a sister clade to the clades of *A.broussonetiae* T.Y. Zhang, W.Q. Chen & M.X. Gao (ex-type, CBS 121455), *A.yali-inficiens* R.G. Roberts (ex-type, CBS 121547), *A.seleniiphila* Wangeline & E.G. Simmons (ex-type, CBS 127671) and *A.lini* P.K. Dey (ex-type, CBS 106.34), namely *A.cunninghamiicola* sp. nov. (Fig. 1). When applying the GCPSR concept to these isolates, the concatenated sequence dataset of nine-loci (ITS, SSU, LSU, GAPDH, RPB2, TEF1, Alt a1, endoPG and OPA10-2) was subjected to the PHI test, and the result showed that no significant recombination was detected amongst these isolates/taxa (Φw = 0.2087) (Fig. 2C). It was a solid support for the proposition that these isolates belonged to five distinct taxa.

### ﻿Taxonomy

Based on morphology and multi-locus sequence data, a total of 48 obtained isolates from Chinese fir were assigned to seven species of *Alternaria*, which represented seven undescribed taxa and were described below.

#### ﻿ Alternaria cunninghamiicola

Taxon classificationFungiPleosporalesPleosporaceae

Lin Huang, Jiao He & D.W. Li sp. nov.

CD5CDB09-EAFE-50CF-A430-EAF00E9161DA

Index Fungorum: IF901036

Fig. 3

##### Holotype.

China, Jiangsu Province, Nanjing City, Dongshanqiao Forest Farm, 31°51'11"N, 118°46'12"E, isolated from leaf spots of *Cunninghamialanceolata*, May 2017, Wen-Li Cui, (holotype: CFCC 59358). Holotype specimen is a living specimen being maintained via lyophilisation at the China Forestry Culture Collection Center (CFCC). Ex-type (DSQ3-2) is maintained at the Forest Pathology Laboratory, Nanjing Forestry University.

##### Etymology.

The specific epithet refers to the genus of the host plant (*Cunninghamialanceolata*).

##### Host/distribution.

From *C.lanceolata* in Dongshanqiao Forest Farm, Nanjing City, Jiangsu Province, China.

##### Description.

Mycelium superficial on the PCA, composed of septate, branched, smooth, thin-walled, pale white to grey hyphae. Conidiophores macronematous, mononematous, solitary, subcylindrical, branched or unbranched, straight or geniculate, thin-walled, 2–10 septate, (18.3–)25.3–68.4(–93.8) × (3.0–)3.3–4.2(–4.8) μm, (mean ± SD = 46.9 ± 21.6 × 3.7 ± 0.5 μm, n = 32), arising mostly at right angles from undifferentiated hyphae, with conspicuous scars after conidia have seceded. Conidiogenous cells apical or subapical, cylindrical, light brown, smooth, (5.2–)7.3–14.0(–18.1) × (2.5–)3.0–4.2(–5.0) μm, (mean ± SD = 10.7 ± 3.3 × 3.6 ± 0.6 μm, n = 45), mono- or polytretic, with conspicuous scars at the loci of sporulating after conidia have seceded. Each conidiogenous locus bears a primary chain of 3–5 conidia with rarely lateral branches or occasionally a sole secondary conidium. Conidia pale brown to brown, shape varied, ovoid or ellipsoid, pyriform or obclavate, usually smooth; conidial bodies (12.2–)18.1–35.4(–51.6) × (7.5–)10.4–15.5(–18.7) μm, (mean ± SD = 26.6 ± 8.6 × 12.9 ± 2.6 μm, n = 53), with 1–5 transverse and 0–2 longitudinal septate. Secondary conidia directly (but rarely) produced by conidia through an inconspicuous apical conidiogenous locus or (commonly) by means of a short apical or lateral secondary conidiophore with 1–2 cells in length. Secondary conidiophores (false beaks) with one or a few conidiogenous loci, (4.5–)5.2–22.5(–32.7) × (2.7–)3.2–4.2(–4.7) μm, (mean ± SD = 13.8 ± 8.7 × 3.7 ± 0.5 μm, n = 31). Beakless conidia mostly with a conical cell at the apex. Chlamydospores not observed.

##### Culture characteristics.

Colonies on PCA incubated at 25 °C in the dark growing at 9.3 ± 0.1 mm/d; aerial hypha cottony, white to pale grey; reverse centre dark green to black; sporulation sparse; diffusible pigment absent.

##### Additional materials examined.

China, Jiangsu Province, Nanjing City, Dongshanqiao Forest Farm, 31°51'11"N, 118°46'12"E, isolated from leaf spots of *Cunninghamialanceolata*, May 2017, Wen-Li Cui, DSQ3-2-1, DSQ3-2-2, DSQ3-2-3, DSQ3-2-4.

##### Notes.

The isolates of *A.cunninghamiicola* were phylogenetically close to *A.broussonetiae* (ex-type, CBS 121455), *A.yali-inficiens* (ex-type, CBS 121547), *A.seleniiphila* (ex-type, CBS 127671) and *A.lini* (ex-type, CBS 106.34) (Fig. 2). Between *A.cunninghamiicola* isolates and *A.broussonetiae* (ex-type, CBS 121455), there were 1/453 differences in Alt a1, 4/510 in ITS and 1/664 in OPA10-2. Between *A.cunninghamiicola* isolates and *A.yali-inficiens* (ex-type, CBS 121547), there were 1/453 differences in Alt a1, 2/499 in GAPDH, 3/510 in ITS and 1/401 in endoPG. Between *A.cunninghamiicola* isolates and *A.seleniiphila* (ex-type, CBS 127671), there were 1/453 differences in Alt a1, 2/499 in GAPDH, 3/510 in ITS, 1/401 in endoPG and 6/757 in RPB2. Between *A.cunninghamiicola* isolates and *A.lini* (ex-type, CBS 106.34), there were 1/453 differences in Alt a1, 2/499 in GAPDH, 4/510 in ITS, 1/887 in LSU, 1/664 in OPA10-2 and 6/757 in RPB2. The PHI analysis showed that there was no significant recombination between *A.cunninghamiicola* isolates and its related species (Φ_w_ = 0.2087) (Fig. 2C). Distinguishing characteristics of this new species and other related species of *Alternaria* spp. are shown in Table 2. Morphologically, conidia in chains of the *A.cunninghamiicola* isolates were less than those of *A.broussonetiae*CBS 121455 (ex-type) (3–5 vs. 8–15 conidia) (Zhang et al. 1999) and *A.yali-inficiens*CBS 121547 (ex-type) (3–5 vs. 8–18 conidia) (Roberts 2005). Conidiophores of the *A.cunninghamiicola* isolates were shorter than those of *A.seleniiphila*CBS 127671 (ex-type) (25.3–68.4 × 3.3–4.2 μm vs. 80–250 × 4–5 μm) (Wangeline and Reeves 2007). Conidia of the *A.cunninghamiicola* isolates were shorter and wider than those of *A.lini*CBS 106.34 (ex-type) (18.1–35.4 × 10.4–15.5 μm vs. 42–60 × 3–7 μm) (Dey 1933). Thus, the phylogenetic and morphological evidence support this fungus being a new species within the *Alternariaalternata* species complex.

**Figure 3.**
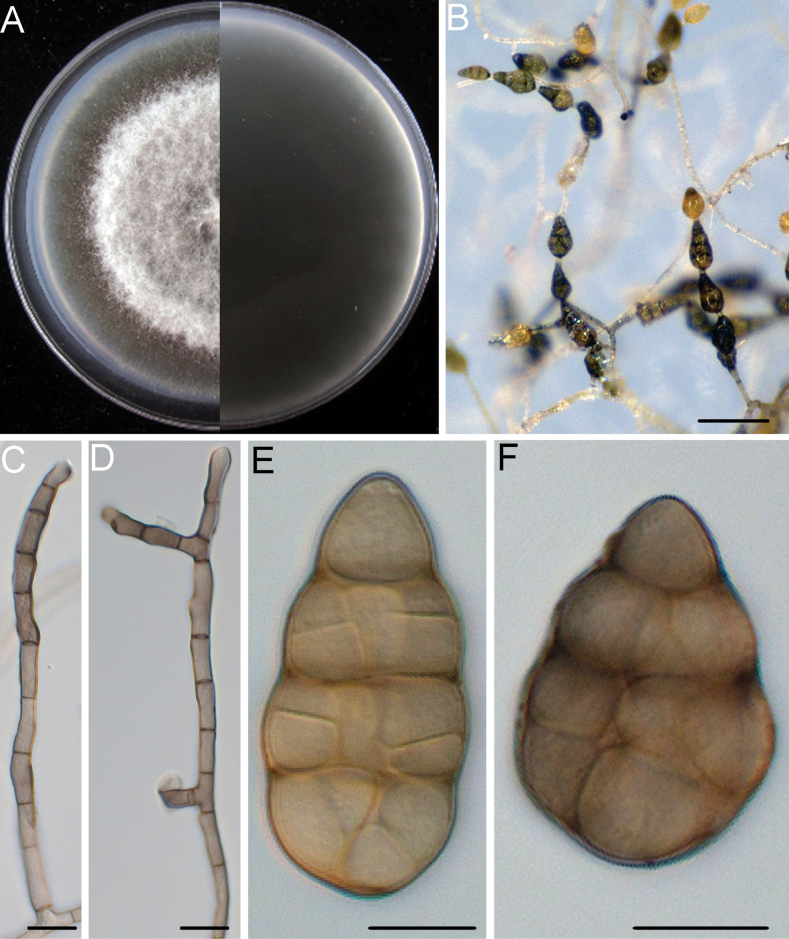
*Alternariacunninghamiicola* (DSQ3-2) **A** colony on PCA after 6 days at 25 °C in the dark **B** sporulation patterns **C, D** conidiophores and conidiogenous cell **E, F** conidium. Scale bars: 50 μm (**B**); 10 μm (**C–F**).

**Table 2.** Distinguishing characteristics of the new species and similar known species of *Alternaria* spp. under growth conditions^a^.

Species	Conidiophores (μm)b	Conidiogenous cells (μm)c	Chain	Size (μm)d	Conidia Transverse septa	Longitudinal or oblique septa	Beak or secondary conidiophores (false beaks) (μm)e	Reference
*Alternariabroussonetiae* (ex-type, CBS 121455)	np	np	8–15 conidia	25–38 × 9–12	5–6	0–1	beakless secondary conidiophore single hyaline cell 3–4 × 3–5 a well-differentiated up to ca 25–50 × 3–4	Zhang et al. (1999)
*A.cinerariae* (ex-epitype, CBS 612.72)	25–196 × 6–11	np	2–5(–9) conidia	18–295 × 8–63	1–14	up to 10	80–159 × 5–9	(Nishikawa and Nakashima 2020)
*A.citri* (ex-epitype, CBS 107.27)	np	np	3–6 conidia	10–22 × 8–15 (in early stages) 25–40 × 15–25 (Mature)	(3–)4–6	one or more	np	(Pierce 1902)
*A.citriarbusti* (ex-type, CBS 102598)	200 × 5	np	5–8 conidia	30–60 × 8–12	6–11	0–1	beakless secondary conidiophores single cell 3–5 × 4 elongate but not filiform extension up to 25–35 × 2–3	(Simmons 1999)
***A.cunninghamiicola* (DSQ3-2)**	**25.3–68.4 × 3.3–4.2**	**7.3–14.0 × 3.0–4.1**	**3–5 conidia**	**18.1–35.4 × 10.4–15.5**	**1–6**	**0–5**	**beakless secondary conidiophores (false beaks) 5.2–22.5 × 3.2–4.2**	**this study**
***A.dongshanqiaoensis* (DSQ2-2)**	**16.4–60.2 × 3.2–4.6**	**5.2–13.7 × 3.5–4.6**	**5–9 conidia**	**21.1–32.9 × 11.4–16.8**	**1–4**	**1–4**	**beakless, secondary conidiophores (false beaks) 2.2–9.4 × 2.8–4.0**	**this study**
***A.hunanensis* (HN43-10-2)**	**18.4–41.8 × 3.7–4.7**	**4.6–9.5 × 3.0–4.5**	**3–7 conidia; one secondary chain of 1–2 conidia.**	**16.7–28.8 × 8.2–12.6**	**1–4**	**0–2**	**beakless, secondary conidiophores (false beaks) 2.9–21.7 × 2.8–4.3**	**this study**
*A.kikuchiana* (ex-holotype, CBS 107.53)	np	np	6–9 conidia	10–70 × 6–22	1–3	1–10	np	(Nishikawa and Nakashima 2019)
***A.kunyuensis* (XXG21)**	**21.4–53.5 × 3.3–4.0**	**5.2–11.1 × 3.2–4.2**	**3–8 conidia; one secondary chain of 2–4 conidia.**	**20.5–29.8 × 9.4–13.5**	**1–5**	**0–3**	**beakless, secondary conidiophores (false beaks) 2.9–20.0 × 2.8–3.9**	**this study**
*A.lini* (ex-type, CBS 106.34)	26–80 × 3–7	np	np	42–60 × 3–7	2–7	1–4	beakless	(Dey 1933)
***A.longqiaoensis***	**19.6–51.0 × 3.3–4.2**	**4.3–9.6 × 2.9–4.5**	**4–8 conidia; 1 to 3 secondary chains of 3–4**	**16.0–28.2 × 7.0–12.6**	**1–5**	**0–2**	**beakless, secondary conidiophores (false beaks) 3.3–11.6 × 2.9–3.9**	**this study**
*A.platycodonis* (ex-type, CBS 121348)	np	np	8–10 conidia	25–45 × 8–12	4–7	0	beaklesssecondary conidiophore single hyaline cell 3–4 × 3–5 well-differentiated up to 20 × 3–4	(Zhang 2003)
*A.rhadina* (ex-type, CBS 595.93)	60–110 × 3–4	np	9–15 conidia 35–45 × 8–9 (narrow ovoid)	4–7	1	20–45 (tapered beak)		
*A.seleniiphila* (ex-type, CBS 127671)	80–250 × 4–5	np	3–6 conidia	20–40 × 8–12	1–7	0–1	beakless secondary conidiophores (false beaks) 3–30 × 3	(Wangeline and Reeves 2007)
***A.shandongensis* (SDHG12)**	**23.6–51.1 × 3.4–4.3**	**4.8–9.6 × 3.2–4.3**	**9–13 conidia**	**20.1–31.2 × 9.3–14.1**	**2–7**	**0–3**	**beakless, secondary conidiophores (false beaks) 2.7–10.3 × 2.3–3.1**	**this study**
*A.tenuissima* (ex-epitype, CBS 620.83)	np	np	6–10 conidia	32–45 × 11–13 (only transverse septa) 32–45 × 14–18 (ovoid muriformly septate)	np	np	narrow-taper beak is near 64(–72)	(Wiltshire 1933)
*A.tomaticola* (ex-epitype, CBS 118814)	50–80 × 3–5	np	10–15 conidia	30–40 × 9–12 (larger conidia)	6–7 (larger)	1–2 (larger)	beakless secondary conidiophores 15–50	(Simmons 2007)
				12–25 × 7–13 (smaller conidia)	1–4 (smaller)	0–1 (smaller)		
*A.vaccinii* (ex-epitype, CBS 118818)	100–200 × 3–4	np	8–10 conidia	15–50 × 7–9	1–8	np	beakless secondary conidiophores 65–150 × 3–4	(Simmons 2007)
***A.xinyangensis* (ZLS1)**	**15.3–54.9 × 3.7–4.8**	**5.3–9.6 × 3.3–4.9**	**2–7 conidia**	**19.9–31.8 × 8.6–12.9**	**1–6**	**1–5**	**beakless, secondary conidiophores (false beaks) 5.3–16.0 × 2.8–4.1**	**this study**
*A.yali-inficiens* (ex-type, CBS 121547)	80–120 × 4–5	np	8–18 conidia	20–30 × 10–12	3–4	1–2	np	(Roberts 2005)

a New species in this study are printed in bold. bcde Dimensions of conidiophores, Conidiogenous cells, conidia, and beaks (μm, mean ± SD for length × width). np: no product.

#### ﻿ Alternaria dongshanqiaoensis

Taxon classificationFungiPleosporalesPleosporaceae

Lin Huang, Jiao He & D.W. Li sp. nov.

C6288EFD-C5F6-5F46-9C54-F8A791DAAE7E

Index Fungorum: IF901037

Fig. 4

##### Holotype.

China, Jiangsu Province, Nanjing City, Dongshanqiao Forest Farm, 31°51'11"N, 118°46'12"E, isolated from leaf spots of *Cunninghamialanceolata*, May 2017, Wen-Li Cui, (holotype: CFCC 59353). Holotype specimen is a living specimen being maintained via lyophilisation at the China Forestry Culture Collection Center (CFCC). Ex-type (DSQ2-2) is maintained at the Forest Pathology Laboratory, Nanjing Forestry University.

##### Etymology.

Epithet is after Dongshanqiao Forest Farm, Nanjing City, Jiangsu Province where the type specimen was collected.

##### Host/distribution.

from *C.lanceolata* in Dongshanqiao Forest Farm, Nanjing City, Jiangsu Province, China.

##### Description.

Mycelium superficial on the PCA, composed of septate, branched, smooth, thin-walled, white to pale brown hyphae. Conidiophores macronematous, mononematous, solitary and relatively short, pale brown, smooth, 1–3 septate, (8.1–)16.4–60.2(–100.5) × (2.4–)3.2–4.6(–5.6) μm, (mean ± SD = 38.3 ± 21.9 × 3.9 ± 0.7 μm, n = 30), arising mostly at right angles from undifferentiated hyphae. Conidiogenous cells apical or subapical, cylindrical, light brown, smooth, (3.8–)5.2–13.7(–20.2) × (2.8–)3.5–4.6(–5.2) μm, (mean ± SD = 9.4 ± 4.2 × 4.0 ± 0.5 μm, n = 36), mono- or di-tretic, with conspicuous scars at the loci of sporulating after conidia have seceded. Each conidiogenous locus bears a primary chain of 5–9 conidia; rarely with lateral branches or occasionally a sole secondary conidium. Conidial bodies brown to dark brown, ellipsoid to obclavate, smooth to verruculose, (16.4–)21.1–32.9(–40.1) × (10.2–)11.4–16.8(–22.2) μm, (mean ± SD = 27.0 ± 5.9 × 14.1 ± 2.7 μm, n = 48), with 1–4 (mostly 3) transverse and 1–4 longitudinal septate. Secondary conidia commonly produced by means of a short apical or lateral secondary conidiophore, but rarely by conidia through an inconspicuous apical conidiogenous locus. Secondary conidiophores (false beaks) at the apical end and median of conidium, short, mostly single-celled, (1.4–)2.2–9.4(–20.0) × (1.9–)2.8–4.0(–5.2) μm, (mean ± SD = 5.8 ± 3.6 × 3.4 ± 0.6 μm, n = 33). Beakless conidia mostly with a conical cell at the apex. Chlamydospores not observed.

**Figure 4.**
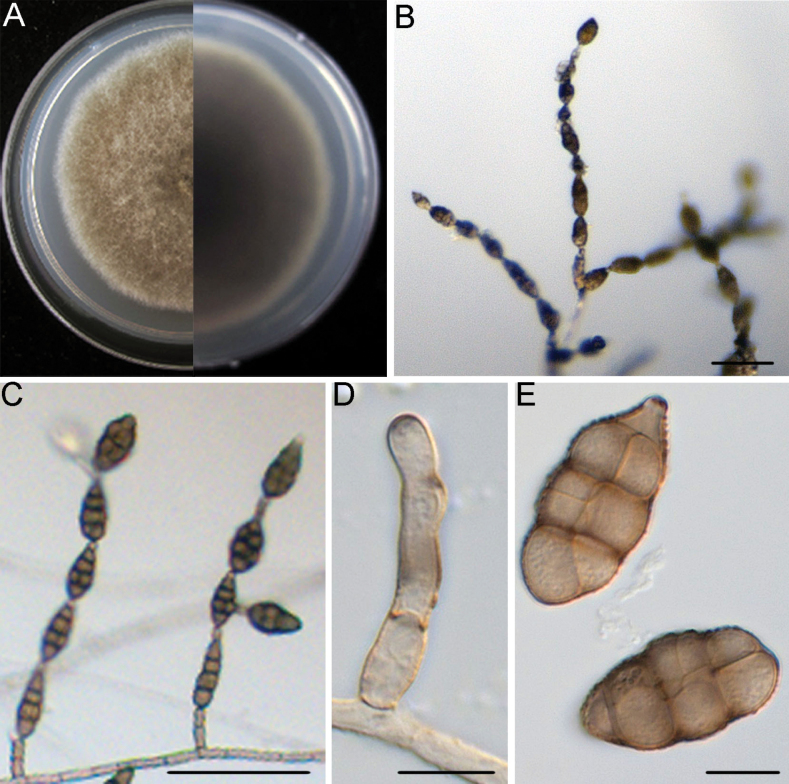
*Alternariadongshanqiaoensis* (DSQ2-2) **A** colony on PCA after 6 days at 25 °C in the dark **B, C** sporulation patterns **D** conidiophore and conidiogenous cell **E** conidia. Scale bars: 50 μm (**B, C**); 10 μm (**D, E**).

##### Culture characteristics.

Colonies on PCA incubated at 25 °C in the dark growing at 7.8 ± 0.2 mm/d; aerial hyphae cottony, greyish-green, with grey margins; reverse centre black, with white margins.

##### Additional materials examined.

China, Jiangsu Province, Nanjing City, Dongshanqiao Forest Farm, 31°51'11"N, 118°46'12"E, isolated from leaf spots of *Cunninghamialanceolata*, May 2017, Wen-Li Cui, DSQ2-2-1, DSQ2-2-2, DSQ2-2-3, DSQ2-2-4; Hunan Province, Yiyang City, Longqiao Town, 28°27'24"N, 112°29'7"E, isolated from leaf spots of *C.lanceolata*, May 2017, Wen-Li Cui, HN43-6-1, HN43-6-1-1, HN43-6-1-2, HN43-6-1-3, HN43-6-1-4.

##### Notes.

The isolates of *A.dongshanqiaoensis* were phylogenetically close to *A.citri* (ex-epitype, CBS 107.27), *A.cinerariae* (ex-epitype, CBS 612.72), *A.kikuchiana* (ex-holotype, CBS 107.53) and *A.tenuissima* (Kunze) Wiltshire (ex-epitype, CBS 620.83) (Fig. 2). Between *A.dongshanqiaoensis* isolates and *A.citri* (ex-epitype, CBS 107.27), there were 2/453 differences in Alt a1, 4/510 in ITS, 2/401 in endoPG, 1/757 in RPB2 and 2/996 in SSU. Between *A.dongshanqiaoensis* isolates and *A.cinerariae* (ex-epitype, CBS 612.72), there were 2/453 differences in Alt a1, 4/510 in ITS, 2/401 in endoPG, 1/757 in RPB2 and 2/996 in SSU. Between *A.dongshanqiaoensis* isolates and *A.kikuchiana* (ex-type, CBS 107.53), there were 2/453 differences in Alt a1, 4/510 in ITS, 8/664 in OPA10-2, 3/401 in endoPG, 2/757 in RPB2 and 2/996 in SSU. Between *A.dongshanqiaoensis* isolates and *A.tenuissima* (ex-epitype, CBS 620.83), there were 1/453 differences in Alt a1, 6/510 in ITS, 8/664 in OPA10-2, 3/401 in endoPG, 1/757 in RPB2 and 6/996 in SSU. The PHI analysis showed that there was no significant recombination between *A.dongshanqiaoensis* isolates and its related species (Φ_w_ = 0.1647) (Fig. 2A). Distinguishing characteristics of this new species and other related species of *Alternaria* spp. are shown in Table 2. Morphologically, conidia in chains of the *A.dongshanqiaoensis* isolates were more than those of *A.citri*CBS 107.27 (ex-epitype) (5–9 conidia vs. 3–6 conidia) (Pierce 1902). Conidia of the *A.dongshanqiaoensis* isolates were significantly different from those of *A.cinerariae*CBS 612.72 (ex-epitype) (21.1–32.9 × 11.4–16.8 μm vs. 18–295 × 8–63 μm) (Nishikawa and Nakashima 2020). Longitudinal septa of conidia of the *A.dongshanqiaoensis* isolates were less than those of *A.kikuchiana*CBS 107.53 (ex-holotype) (1–4 vs. 1–10 longitudinal or oblique septa) (Nishikawa and Nakashima 2019). Conidia of the *A.dongshanqiaoensis* isolates were different from those of *A.tenuissima*CBS 620.83 (ex-epitype) (beakless vs. with a narrow-taper beak) (Wiltshire 1933). In conclusion, the phylogenetic and morphological evidence support this fungus as being a new species within the *Alternariaalternata* species complex.

#### ﻿ Alternaria hunanensis

Taxon classificationFungiPleosporalesPleosporaceae

Lin Huang, Jiao He & D.W. Li sp. nov.

496835B3-4D40-5986-90AF-9AE8BF504BBE

Index Fungorum: IF901038

Fig. 5

##### Holotype.

China, Hunan Province, Yiyang City, Longqiao Town, 28°27'24"N, 112°29'7"E, isolated from leaf spots of *Cunninghamialanceolata*, May 2017, Wen-Li Cui, (holotype: CFCC 59356). Holotype specimen is a living specimen being maintained via lyophilisation at the China Forestry Culture Collection Center (CFCC). Ex-type (HN43-10-2) is maintained at the Forest Pathology Laboratory, Nanjing Forestry University.

##### Etymology.

Epithet is after Longqiao Town, Yiyang City, Hunan Province where the type specimen was collected.

##### Host/distribution.

From *C.lanceolata* in Longqiao Town, Yiyang City, Hunan Province, China.

##### Description.

Mycelium superﬁcial on the PCA medium, composed of septate, branched, smooth, thin-walled, white to light brown hyphae. Conidiophores macronematous, mononematous, solitary, subcylindrical, branched or unbranched, straight or geniculate, (12.7–)18.4–41.8(–65.0) × (2.5–)3.3–4.7(–5.2) μm, (mean ± SD = 30.1 ± 11.7 × 4.0 ± 0.7 μm, n = 45). Each conidiogenous locus bears a primary chain of 3–7 conidia; each chain usually has a secondary chain of 1–2 conidia. Conidiogenous cells apical or subapical, cylindrical, light brown, smooth, (2.9–)4.6–9.5(–13.6) × (1.8–)3.0–4.5(–6.3) μm, (mean ± SD = 7.0 ± 2.5 × 3.8 ± 0.8 μm, n = 46), mono- or polytretic. Newly developed conidia subhyaline or pale greyish, ellipsoidal or subacute, thin-walled, with few or no protuberance. Mature conidia pale brown to brown, ovoid or ellipsoid to long-ellipsoid, pyriform, usually smooth. Conidial bodies (10.0–)16.7–28.8(–39.3) × (5.9–)8.2–12.6(–14.8) μm, (mean ± SD = 22.7 ± 6.0 × 10.4 ± 2.2 μm, n = 49), with 1–4 transverse and 0–2 longitudinal septa. Secondary conidia commonly produced by means of a short apical or lateral secondary conidiophore, but rarely by conidia through an inconspicuous apical conidiogenous locus. Secondary conidiophores (false beaks) at the apical end and median of conidium, short, mostly single-celled, (2.8–)2.9–21.7(–41.7) × (2.5–)2.8–4.3(–6.2) μm, (mean ± SD = 12.3 ± 9.4 × 3.5 ± 0.7 μm, n = 37). Conidial beakless mostly with a conical cell at the apex. Chlamydospores not observed.

**Figure 5.**
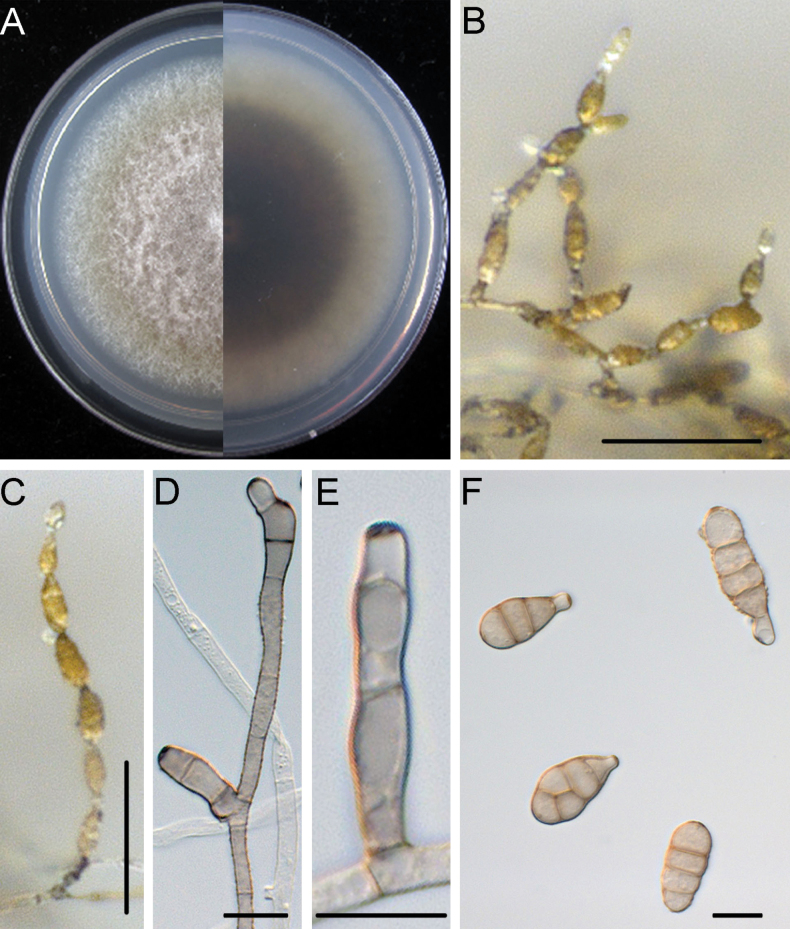
*Alternariahunanensis* (HN43-10-2) **A** colony on PCA after 6 days at 25 °C in the dark **B, C** sporulation patterns **D, E** conidiophores and conidiogenous cells **F** conidia. Scale bars: 50 μm (**B, C**); 10 μm (**D–F**).

##### Culture characteristics.

Colonies on PCA incubated at 25 °C in the dark growing at 7.8 ± 0.1 mm/d; aerial hypha cottony, pale gray to greyish-green, with white to pale grey margins; reverse centre brownish to dark green with pale grey margins; sporulation sparse; diffusible pigment absent.

##### Additional materials examined.

China, Hunan Province, Yiyang City, Longqiao Town, 28°27'24"N, 112°29'7"E, isolated from leaf spots of *Cunninghamialanceolata*, May 2017, Wen-Li Cui, HN43-10-2-1, HN43-10-2-2, HN43-10-2-3, HN43-10-2-4.

##### Notes.

The isolates of *A.hunanensis* were phylogenetically close to *A.longqiaoensis* (this study, HN43-14), *A.vaccinii* (ex-type, CBS 118818), *A.platycodonis* (ex-type, CBS 121348), *A.rhadina* E.G. Simmons (ex-type, CBS 595.93), *A.citriarbusti* (ex-type, CBS 102598) and *A.tomaticola* (ex-type, CBS 118814) (Fig. 2). Between *A.hunanensis* isolates and *A.longqiaoensis* HN43-14, there were 2/453 differences in Alt a1, 3/510 in ITS, 2/401 in endoPG, 2/757 in RPB2 and 18/996 in SSU. Between *A.hunanensis* isolates and *A.vaccinii* (ex-type, CBS 118818), there were 4/453 differences in Alt a1, 2/499 in GAPDH, 3/510 in ITS and 3/401 in endoPG. Between *A.hunanensis* isolates and *A.platycodonis* (ex-type, CBS 121348), there were 1/453 differences in Alt a1, 2/499 in GAPDH, 3/510 in ITS and 2/401 in endoPG. Between *A.hunanensis* isolates and *A.rhadina* (ex-type, CBS 595.93), there were 1/453 differences in Alt a1, 2/499 in GAPDH, 3/510 in ITS and 2/401 in endoPG. Between *A.hunanensis* isolates and *A.citriarbusti* (ex-type, CBS 102598), there were 1/453 differences in Alt a1, 2/499 in GAPDH, 3/510 in ITS and 2/401 in endoPG. Between *A.hunanensis* isolates and *A.tomaticola* (ex-type, CBS 118814), there were 3/453 differences in Alt a1, 2/499 in GAPDH, 3/510 in ITS and 2/401 in endoPG. The PHI analysis showed that there was no significant recombination between *A.hunanensis* isolates and its related species (Φ_w_ = 0.3502) (Fig. 2B). Distinguishing characteristics of this new species and other morphologically related species of *Alternaria* spp. are shown in Table 2. Morphologically, sporulation patterns of the *A.hunanensis* isolates were different from those of *A.longqiaoensis* HN43-14 (one secondary chain of 1–2 conidia vs. 1–3 further branching chains (secondary, tertiary and quaternary chains) of 3–4 conidia). Conidia in chains of the *A.hunanensis* isolates were less than those of *A.vaccinii*CBS 118818 (ex-type) (3–7 vs. 8–10 conidia) (Simmons 2007), *A.platycodonis*CBS 121348 (ex-type) (3–7 vs. 8–10 conidia) (Zhang 2003), *A.rhadina*CBS 595.93 (ex-type) (3–7 vs. 9–15 conidia) (Simmons 1993) and *A.tomaticola*CBS 118814 (ex-type) (3–7 vs. 10–15 conidia) (Simmons 2007). Transverse septa of conidia of the *A.hunanensis* isolates were less than those of *A.citriarbusti*CBS 102598 (ex-type) (1–4 vs. 6–11 transverse septa) (Simmons 1999). Thus, the phylogenetic and morphological evidence supports this fungus as being a new species within the *Alternariaalternata* species complex.

#### ﻿ Alternaria kunyuensis

Taxon classificationFungiPleosporalesPleosporaceae

Lin Huang, Jiao He & D.W. Li sp. nov.

4544FD41-3FA6-56E9-B898-ECEF2329C83C

Index Fungorum: IF901039

Fig. 6

##### Holotype.

China, Shandong Province, Yantai City, Kunyu Mountain, 37°15'22"N, 121°46'05"E, isolated from leaf spots of *Cunninghamialanceolata*, May 2017, Wen-Li Cui, (holotype: CFCC 59355). Holotype specimen is a living specimen being maintained via lyophilisation at the China Forestry Culture Collection Center (CFCC). Ex-type (XXG21) is maintained at the Forest Pathology Laboratory, Nanjing Forestry University.

##### Etymology.

Epithet is after Kunyu Mountain, Yantai City, Shandong Province where the type specimen was collected.

##### Host/distribution.

From *C.lanceolata* in Kunyu Mountain, Yantai City, Shandong Province, China.

##### Description.

Mycelium superﬁcial on the PCA medium, composed of septate, branched, smooth, thin-walled, colourless to pale brown hyphae. Conidiophores short to long, straight or geniculate, simple or branched, pale brown, 1–5 septate, with one or several apical conidiogenous loci, (17.0–)21.4–53.5(–79.2) × (3.0–)3.3–4.0(–4.6) μm, (mean ± SD = 37.4 ± 16.0 × 3.6 ± 0.4 μm, n = 33). Each conidiogenous locus bears a primary chain of 3–8 conidia; each chain usually has one secondary chain of 2–4 conidia. Conidiogenous cells apical or subapical, cylindrical, light brown, smooth, (3.6–)5.2–11.1(–14.7) × (2.5–)3.2–4.2(–4.7) μm, (mean ± SD = 8.1 ± 2.9 × 3.7 ± 0.5 μm, n = 37), mono- or polytretic. Conidia ovoid to ellipsoid, pyriform, pale brown to brown, usually smooth; conidial bodies (16.1–)20.5–29.8(–36.3) × (7.7–)9.4–13.5(–15.8) μm, (mean ± SD = 25.1 ± 4.6 × 11.5 ± 2.0 μm, n = 43), 1–5 transverse and 0–3 longitudinal septate, slightly constricted at the median. Some septa darkened. Secondary conidia commonly produced via a short apical or lateral secondary conidiophore, but rarely by conidia through an inconspicuous apical conidiogenous locus. Secondary conidiophores (false beaks) at the apical end and median of conidium, short or long, multicellular or single cell, (2.9–)2.9–20.0(–37.3) × (2.3–)2.8–3.9(–4.6) μm, (mean ± SD = 11.5 ± 8.5 × 3.3 ± 0.6 μm, n = 33). Conidial beakless mostly with a conical cell at the apex. Chlamydospores not observed.

**Figure 6.**
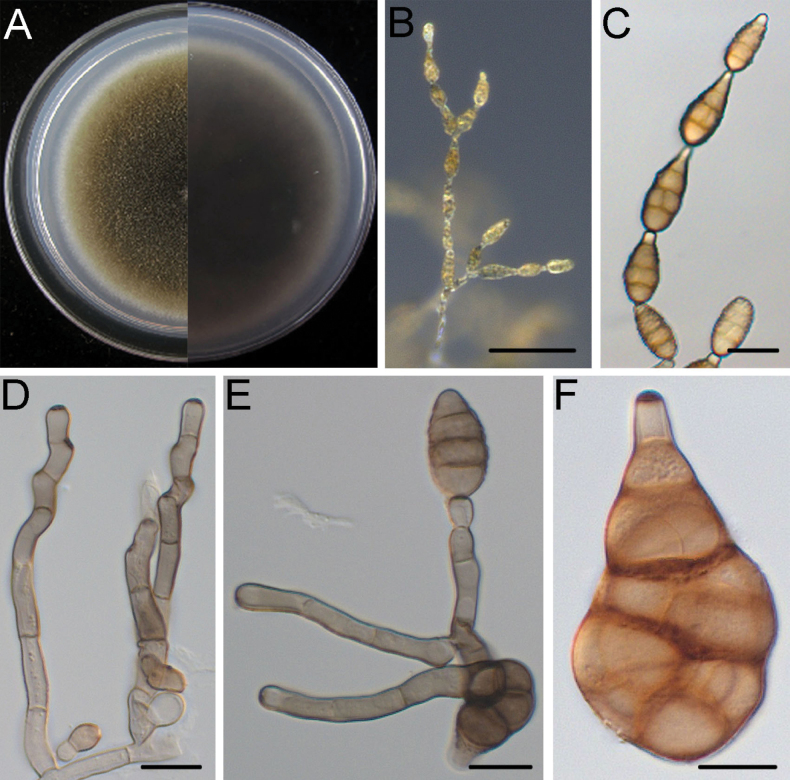
*Alternariakunyuensis* (XXG21) **A** colony on PCA after 6 days at 25 °C in the dark **B, C** sporulation patterns **D** conidiophores bear conidiogenous cells **E** secondary conidiophores, conidiogenous cells and conidia **F** conidium. Scale bars: 50 μm (**B**); 10 μm (**C–F**).

##### Culture characteristics.

Colonies on PCA incubated at 25 °C in the dark growing at 7.5 ± 0.2 mm/d; aerial hypha sparse, olive green to dark green; reverse centre grey; sporulation abundant; diffusible pigment absent.

##### Additional materials examined.

China, Shandong Province, Yantai City, Kunyu Mountain, 37°15'22"N, 121°46'05"E, isolated from leaf spots of *Cunninghamialanceolata*, May 2017, Wen-Li Cui, XXG12-2, XXG22, XXG26-2, XXG30, XXG31.

##### Notes.

The isolates of *A.kunyuensis* were phylogenetically close to *A.hunanensis* (this study, HN43-10-2), *A.longqiaoensis* (this study, HN43-14), *A.vaccinii* (ex-type, CBS 118818), *A.platycodonis* (ex-type, CBS 121348), *A.rhadina* (ex-type, CBS 595.93), *A.citriarbusti* (ex-type, CBS 102598) and *A.tomaticola* (ex-type, CBS 118814) (Fig. 2). Between *A.kunyuensis* isolates and *A.hunanensis* HN43-10-2, there were 2/453 differences in Alt a1, 1/510 in ITS, 1/664 in OPA10-2, 5/401 in endoPG, 4/757 in RPB2, 1/996 in SSU and 3/293 in TEF1. Between *A.kunyuensis* isolates and *A.longqiaoensis* HN43-14, there were 3/453 differences in Alt a1, 2/510 in ITS, 1/664 in OPA10-2, 3/401 in endoPG, 6/757 in RPB2, 19/996 in SSU and 3/293 in TEF1. Between *A.kunyuensis* isolates and *A.vaccinii*CBS 118818 (ex-type), there were 5/453 differences in Alt a1, 2/499 in GAPDH, 3/510 in ITS, 1/664 in OPA10-2, 4/401 in endoPG, 4/757 in RPB2, 1/996 in SSU and 3/293 in TEF1. Between *A.kunyuensis* isolates and *A.platycodonis*CBS 121348 (ex-type), there were 2/453 differences in Alt a1, 2/499 in GAPDH, 3/510 in ITS, 1/664 in OPA10-2, 3/401 in endoPG, 4/757 in RPB2, 1/996 in SSU and 3/293 in TEF1. Between *A.kunyuensis* isolates and *A.rhadina*CBS 595.93 (ex-type), there were 2/453 differences in Alt a1, 2/499 in GAPDH, 3/510 in ITS, 1/664 in OPA10-2, 3/401 in endoPG, 4/757 in RPB2, 1/996 in SSU and 3/293 in TEF1. Between *A.kunyuensis* isolates and *A.citriarbusti*CBS 102598 (ex-type), there were 2/453 differences in Alt a1, 3/510 in ITS, 1/664 in OPA10-2, 3/401 in endoPG, 4/757 in RPB2, 1/996 in SSU and 3/293 in TEF1. Between *A.kunyuensis* isolates and *A.tomaticola*CBS 118814 (ex-type), there were 4/453 differences in Alt a1, 3/510 in ITS, 1/664 in OPA10-2, 3/401 in endoPG, 4/757 in RPB2, 1/996 in SSU and 3/293 in TEF1. The PHI analysis showed that there was no significant recombination between *A.kunyuensis* isolates and its related species (Φ_w_ = 0.3502) (Fig. 2B). Distinguishing characteristics of this new species and other related species of *Alternaria* spp. are shown in Table 2. Morphologically, sporulation patterns of the *A.kunyuensis* isolates were different from those of *A.hunanensis* HN43-10-2 (one secondary chain of 2–4 conidia vs. one secondary chain of 1–2 conidia.) and *A.longqiaoensis* HN43-14 (one secondary chain of 2–4 conidia vs. 1–3 branching chains of 3–4 conidia). Conidia in chains of the *A.kunyuensis* isolates were less than those of *A.vaccinii*CBS 118818 (ex-type) (3–8 conidia vs. 8–10 conidia) (Simmons 2007), *A.platycodonis*CBS 121348 (ex-type) (3–8 conidia vs. 8–10 conidia) (Zhang 2003) *A.rhadina*CBS 595.93 (ex-type) (3–8 conidia vs. 9–15 conidia) (Simmons 1993) and *A.tomaticola*CBS 118814 (ex-type) (3–8 conidia vs. 10–15 conidia) (Simmons 2007). Transverse septa of conidia of the *A.kunyuensis* isolates were less than those of *A.citriarbusti*CBS 102598 (ex-type) (1–5 transverse septa vs. 6–11 transverse septa) (Simmons 1999). Thus, the phylogenetic and morphological evidence supports this fungus being as a new species within the *Alternariaalternata* species complex.

#### ﻿ Alternaria longqiaoensis

Taxon classificationFungiPleosporalesPleosporaceae

Lin Huang, Jiao He & D.W. Li sp. nov.

EF378BBF-1B9F-5C76-B9C6-15D3F3144ED3

Index Fungorum: IF901040

Fig. 7

##### Holotype.

China, Hunan Province, Yiyang City, Longqiao Town, 28°27'24"N, 112°29'7"E, isolated from leaf spots of *Cunninghamialanceolata*, May 2017, Wen-Li Cui, (holotype: CFCC 59357). Holotype specimen is a living specimen being maintained via lyophilisation at the China Forestry Culture Collection Center (CFCC). Ex-type (HN43-14) is maintained at the Forest Pathology Laboratory, Nanjing Forestry University.

##### Etymology.

Epithet is after Longqiao Town, Yiyang City, Hunan Province where the type specimen was collected.

##### Host/distribution.

from *C.lanceolata* in Longqiao Town, Yiyang City, Hunan Province, China.

##### Description.

Mycelium superﬁcial on the PCA medium, composed of septate, branched, smooth, thin-walled, pale brown to brown hyphae. Conidiophores macronematous, mononematous, solitary, subcylindrical, unbranched or barely branched, straight or geniculate, 2–4 septa, (4.7–) 19.6–51.0 (–66.3) × (2.9–)3.3–4.2(–4.8) μm, (mean ± SD = 35.3 ± 15.7 × 3.8 ± 0.5 μm, n = 39). Each conidiogenous locus bears a primary chain of 4–8 conidia; each chain usually has 1–3 secondary chains of 3–4 conidia. Conidiogenous cells apical or subapical, cylindrical, light brown, smooth, (2.8–)4.3–9.6(–17.4) × (2.3–)2.9–4.5(–5.8) μm, (mean ± SD = 7.0 ± 2.7 × 3.7 ± 0.8 μm, n = 45), mono- or polytretic. Conidia pale brown to brown, ovoid or ellipsoid to long-ellipsoid, pyriform, smooth or verruculose. Conidial bodies (11.0–)16.0–28.2(–40.2) × (6.1–)7.0–12.6(–20.8) μm, (mean ± SD = 22.1 ± 6.1 × 9.8 ± 2.8 μm, n = 48), with 1–5 transverse and 0–2 longitudinal septate. Secondary conidia commonly produced via a short lateral secondary conidiophore, but rarely by conidia through an inconspicuous apical conidiogenous locus. Apically or laterally formed secondary conidiophores (false beaks) with one or several conidiogenous loci, short, mostly single-celled, (3.5–)3.3–11.6(–19.7) × (2.8–)2.9–3.9(–4.8) μm, (mean ± SD = 7.5 ± 4.2 × 3.4 ± 0.5 μm, n = 33). Conidial beakless mostly with a conical cell at the apex. Chlamydospores not observed.

**Figure 7.**
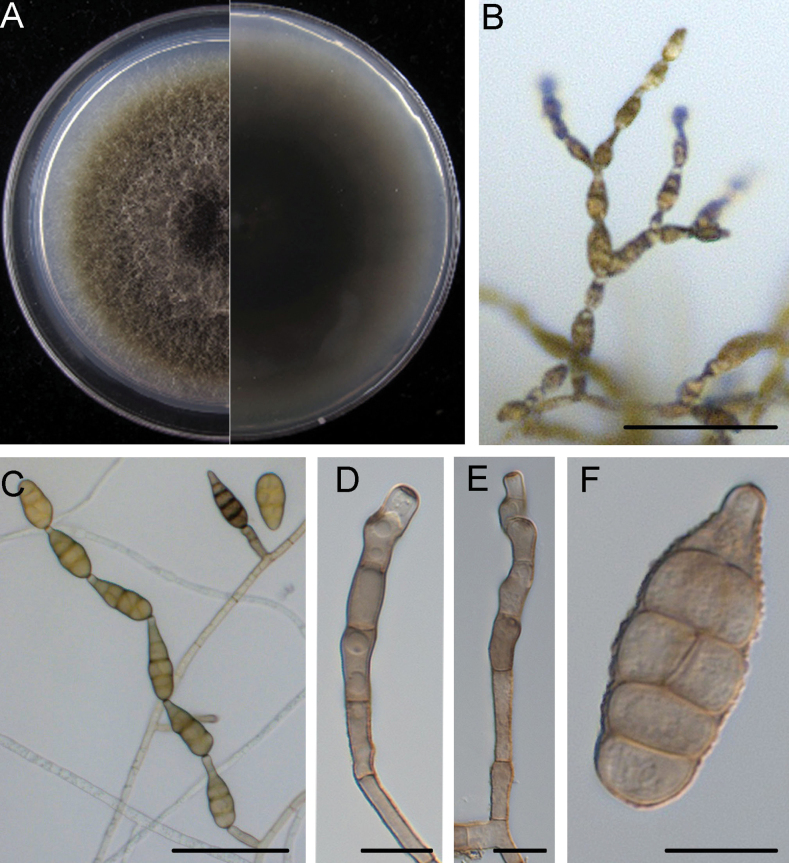
*Alternarialongqiaoensis* (HN43-14) **A** colony on PCA after 6 days at 25 °C in the dark **B, C** sporulation patterns **D, E** conidiophore and conidiogenous cells **F** conidium. Scale bars: 50 μm (**B, C**); 10 μm (**D–F**).

##### Culture characteristics.

Colonies on PCA incubated at 25 °C in the dark growing at 8.3 ± 0.4 mm/d; aerial hypha cottony, dark green to black, with pale green margins; reverse centre black with pale grey margins; sporulation abundant; diffusible pigment absent.

##### Additional materials examined.

China, Hunan Province, Yiyang City, Longqiao Town, 28°27'24"N, 112°29'7"E, isolated from leaf spots of *Cunninghamialanceolata*, May 2017, Wen-Li Cui, HN43-14-1, HN43-14-2, HN43-14-3.

##### Notes.

The isolates of *A.longqiaoensis* were phylogenetically close to *A.vaccinii* (ex-type, CBS 118818), *A.platycodonis* (ex-type, CBS 121348), *A.rhadina* (ex-type, CBS 595.93), *A.citriarbusti* (ex-type, CBS 102598) and *A.tomaticola* (ex-type, CBS 118814) (Fig. 2). Between *A.longqiaoensis* isolates and *A.vaccinii*CBS 118818 (ex-type), there were 4/453 differences in Alt a1, 2/499 in GAPDH, 4/510 in ITS, 1/401 in endoPG, 2/757 in RPB2 and 18/996 in SSU. Between *A.longqiaoensis* isolates and ex-type of *A.platycodonis*CBS 121348, there were 1/453 differences in Alt a1, 2/499 in GAPDH, 4/510 in ITS, 2/757 in RPB2 and 18/996 in SSU. Between *A.longqiaoensis* isolates and *A.rhadina*CBS 595.93 (ex-type), there were 1/453 differences in Alt a1, 2/499 in GAPDH, 4/510 in ITS, 2/757 in RPB2 and 18/996 in SSU. Between *A.longqiaoensis* isolates and *A.citriarbusti*CBS 102598 (ex-type), there were 1/453 differences in Alt a1, 4/510 in ITS, 2/757 in RPB2 and 18/996 in SSU. Between *A.longqiaoensis* isolates and *A.tomaticola*CBS 118814 (ex-type), there were 3/453 differences in Alt a1, 4/510 in ITS, 2/757 in RPB2 and 18/996 in SSU. The PHI analysis showed that there was no significant recombination between *A.longqiaoensis* isolates and its related species (Φ_w_ = 0.3502) (Fig. 2B). Distinguishing characteristics of this new species and other morphologically-related species of *Alternaria* spp. are shown in Table 2. Morphologically, conidia in chains of the *A.longqiaoensis* isolates were less than those of *A.vaccinii*CBS 118818 (ex-type) (4–8 conidia vs. 8–10 conidia) (Simmons 2007), *A.platycodonis*CBS 121348 (ex-type) (4–8 conidia vs. 8–10 conidia) (Zhang 2003) *A.rhadina*CBS 595.93 (ex-type) (4–8 conidia vs. 9–15 conidia) (Simmons 1993) and *A.tomaticola*CBS 118814 (ex-type) (4–8 conidia vs. 10–15 conidia) (Simmons 2007). Transverse septa of conidia of the *A.longqiaoensis* isolates were less than those of *A.citriarbusti*CBS 102598 (ex-type) (1–5 vs. 6–11 transverse septa) (Simmons 1999). Thus, the phylogenetic and morphological evidence supports this fungus as being a new species within the *Alternariaalternata* species complex.

#### ﻿ Alternaria shandongensis

Taxon classificationFungiPleosporalesPleosporaceae

Lin Huang, Jiao He & D.W. Li sp. nov.

6214D26A-5725-5321-9BB3-C5AA4938E345

Index Fungorum: IF901041

Fig. 8

##### Holotype.

China, Shandong Province, Yantai City, Penglai District, Hougou village, 37°27'32"N, 120°46'48"E, isolated from leaf spots of *Cunninghamialanceolata*, May 2017, Wen-Li Cui, (holotype: CFCC 59354). Holotype specimen is a living specimen being maintained via lyophilisation at the China Forestry Culture Collection Center (CFCC). Ex-type (SDHG12) is maintained at the Forest Pathology Laboratory, Nanjing Forestry University.

##### Etymology.

Epithet is after Shandong Province where the type specimen was collected.

##### Host/distribution.

From *C.lanceolata* in Hougou village, Penglai District, Yantai City, Shandong Province, China.

##### Description.

Mycelium superﬁcial on the PCA medium, composed of septate, branched, smooth, thin-walled, pale brown hyphae. Conidiophores solitary, emerging from aerial or creeping hyphae, straight or geniculate, simple or branched, with one or several apical conidiogenous loci, 1–5 septate, variable in length, (16.8–)23.6–51.1(–68.8) × (3.0–)3.4–4.3(–5.0) μm, (mean ± SD = 37.3 ± 13.8 × 3.8 ± 0.4 μm, n = 35). Each conidiogenous locus bears a primary chain of 9–13 conidia; each primary chain usually has 1–3 lateral branches (secondary chains) of 1–2 conidia. Conidiogenous cells apical or subapical, cylindrical, light brown, smooth, (3.9–)4.8–9.6(–17.3) × (2.5–)3.2–4.3(–4.8) μm, (mean ± SD = 7.2 ± 2.4 × 3.7 ± 0.6 μm, n = 46), mono- or polytretic. Conidial bodies ovoid to ellipsoid, brown to dark brown, (14.8–)20.1–31.2(–51.5) × (7.5–)9.3–14.1(–17.0) μm, (mean ± SD = 25.6 ± 5.6 × 11.7 ± 2.4 μm, n = 66), with 2–7 transverse and 0–3 longitudinal septa, mostly smooth to occasionally roughened. Secondary conidia commonly produced via a short lateral secondary conidiophore. Secondary conidiophores (false beaks) at the apical end and median of conidium, short, mostly single-celled, (2.9–)2.7–10.3(–23.5) μm × (2.0–)2.3–3.1(–3.7) μm, (mean ± SD = 6.5 ± 3.9 μm × 2.7 ± 0.4 μm, n = 34). Conidial beakless mostly with a conical cell at the apex. Chlamydospores not observed.

**Figure 8.**
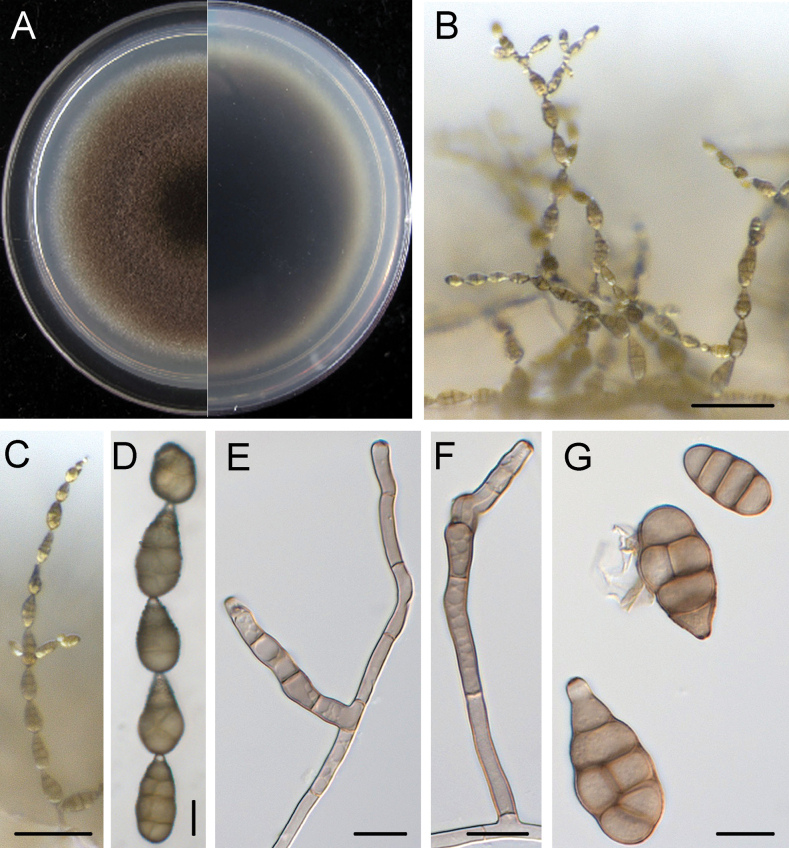
*Alternariashandongensis* (SDHG12) **A** colony on PCA after 6 days at 25 °C in the dark **B–D** sporulation patterns **E, F** conidiophores and conidiogenous cells **G** conidia. Scale bars: 50 μm (**B, C**); 10 μm (**D–G**).

##### Culture characteristics.

Colonies on PCA incubated at 25 °C in the dark growing at 7.6 ± 0.7 mm/d; aerial hypha sparse, dark green to black; reverse centre grey, sporulation abundant; diffusible pigment absent.

##### Additional materials examined.

China, Shandong Province, Yantai City, Penglai District, Hougou village, 37°27'32"N, 120°46'48"E, isolated from leaf spots of *Cunninghamialanceolata*, May 2017, Wen-Li Cui, SDHG12-1, SDHG12-2, SDHG12-3, SDHG12-4; China, Fujian Province, Longyan City, Lianfeng Town, 25°09'27"N, 117°01'50"E, isolated from leaf spots of *C.lanceolata*, May 2017, Wen-Li Cui, LY15.

##### Notes.

The isolates of *A.shandongensis* were phylogenetically close to *A.kunyuensis* (this study, XXG21), *A.hunanensis* (this study, HN43-10-2), *A.longqiaoensis* (this study, HN43-14), *A.vaccinii* (ex-type, CBS 118818), *A.platycodonis* (ex-type, CBS 121348), *A.rhadina* (ex-type, CBS 595.93), *A.citriarbusti* (ex-type, CBS 102598) and *A.tomaticola* (ex-type, CBS 118814) (Fig. 2). Between *A.shandongensis* isolates and *A.kunyuensis* XXG21, there were 1/453 differences in Alt a1, 2/499 in GAPDH, 1/664 in OPA10-2, 5/757 in RPB2, 1/996 in SSU and 3/293 in TEF1. Between *A.shandongensis* isolates and *A.hunanensis* HN43-10-2, there were 1/453 differences in Alt a1, 2/499 in GAPDH, 1/510 in ITS, 5/401 in endoPG and 1/757 in RPB2. Between *A.shandongensis* isolates and *A.longqiaoensis* HN43-14, there were 3/453 differences in Alt a1, 2/499 in GAPDH, 2/510 in ITS, 3/401 in endoPG, 1/757 in RPB2 and 18/996 in SSU. Between *A.shandongensis* isolates and *A.vaccinii*CBS 118818 (ex-type), there were 5/453 differences in Alt a1, 4/499 in GAPDH, 3/510 in ITS, 4/401 in endoPG and 1/757 in RPB2. Between *A.shandongensis* isolates and *A.platycodonis*CBS 121348 (ex-type), there were 2/453 differences in Alt a1, 4/499 in GAPDH, 3/510 in ITS, 3/401 in endoPG and 1/757 in RPB2. Between *A.shandongensis* isolates and *A.rhadina*CBS 595.93 (ex-type), there were 2/453 differences in Alt a1, 4/499 in GAPDH, 3/510 in ITS, 3/401 in endoPG and 1/757 in RPB2. Between *A.shandongensis* isolates and *A.citriarbusti*CBS 102598 (ex-type), there were 2/453 differences in Alt a1, 2/499 in GAPDH, 3/510 in ITS, 3/401 in endoPG and 1/757 in RPB2. Between *A.shandongensis* isolates and *A.tomaticola*CBS 118814 (ex-type), there were 4/453 differences in Alt a1, 2/499 in GAPDH, 3/510 in ITS, 3/401 in endoPG and 1/757 in RPB2. The PHI analysis showed that there was no significant recombination between *A.shandongensis* isolates and its related species (Φ_w_ = 0.3502) (Fig. 2B). Distinguishing characteristics of this new species and their related species of *Alternaria* are shown in Table 2. Morphologically, conidia in chains of the *A.shandongensis* isolates were more than those of *A.kunyuensis* XXG21 (9–13 conidia vs. 6–8 conidia), *A.hunanensis* HN43-10-2 (9–13 conidia vs. 3–7 conidia), *A.longqiaoensis* HN43-14 (9–13 conidia vs. 4–8 conidia), *A.citriarbusti*CBS 102598 (ex-type) (9–13 conidia vs. 5–8 conidia) (Simmons 1999) and *A.platycodonis*CBS 121348 (ex-type) (9–13 conidia vs. 8–10 conidia) (Zhang 2003). Conidiophores of the *A.shandongensis* isolates were significantly shorter than those of *A.vaccinii*CBS 118818 (ex-type) (23.6–51.1 × 3.4–4.3 μm vs. 100–200 × 3–4 μm) (Simmons 2007), *A.rhadina*CBS 595.93 (ex-type) (23.6–51.1 × 3.4–4.3 μm vs. 60–110 × 3–4 μm) (Simmons 1993), *A.citriarbusti*CBS 102598 (ex-type) (23.6–51.1 × 3.4–4.3 μm vs. 200 × 5 μm) (Simmons 1999) and *A.tomaticola*CBS 118814 (ex-type) (23.6–51.1 × 3.4–4.3 μm vs. 50–80 × 3–5 μm) (Simmons 2007). In conclusion, the phylogenetic and morphological evidence supports this fungus as being a new species within the *Alternariaalternata* species complex.

#### ﻿ Alternaria xinyangensis

Taxon classificationFungiPleosporalesPleosporaceae

Lin Huang, Jiao He & D.W. Li sp. nov.

32905332-3E80-5CE9-A233-A601C7A1D8B2

Index Fungorum: IF901042

Fig. 9

##### Holotype.

China, Henan Province, Xinyang City, Zhenlei Mountain, 32°04'51"N, 114°07'23"E, isolated from leaf spots of *Cunninghamialanceolata*, May 2017, Wen-Li Cui, (holotype: CFCC 59352). Holotype specimen is a living specimen being maintained via lyophilisation at the China Forestry Culture Collection Center (CFCC). Ex-type (ZLS1) is maintained at the Forest Pathology Laboratory, Nanjing Forestry University.

##### Etymology.

Epithet is after Xinyang City where the type specimen was collected.

##### Host/distribution.

From *C.lanceolata* in Zhenlei Mountain, Xinyang City, Henan Province, China.

##### Description.

Mycelium superﬁcial on the PCA, composed of septate, branched, smooth, thin-walled, white to light brown hyphae. Conidiophores macronematous, mononematous, produced laterally or terminally on the hyphae, cylindrical, erect or ascending, simple or branched, geniculate, pale brown to dark brown, smooth, 1–7 septate, (9.4–)15.3–54.9(–80.4) × (2.9–)3.7–4.8(–5.2) μm, (mean ± SD = 35.1 ± 19.8 × 4.2 ± 0.6 μm, n = 40). Conidiogenous cells apical or subapical, cylindrical, brown, smooth, (3.9–)5.3–9.6(–12.9) × (2.4–)3.3–4.9(–5.5) μm, (mean ± SD = 7.5 ± 2.2 × 4.1 ± 0.8 μm, n = 39), mono- or polytretic, with conspicuous scars after conidia have seceded. Each conidiogenous locus bears a primary chain of 2–7 conidia; each primary chain usually has 1–3 branching chains of 1–3 conidia. Newly-developed conidia subhyaline or pale greyish, ellipsoidal or subacute, thin-walled, 1–3 septate, with few or no protuberance. Mature conidia brown to dark chocolate–brown, spheroidal or ellipsoid to long-ellipsoid, with 1–6 transverse septa and 1–5 longitudinal or oblique septa, (13.8–)19.9–31.8(–37.6) × (6.9–)8.6–12.9(–17.5) μm, (mean ± SD = 25.9 ± 6.0 × 10.7 ± 2.1 μm, n = 37) in size. Secondary conidia commonly produced by means of a short apical or lateral secondary conidiophore, but rarely by conidia through an inconspicuous apical conidiogenous locus. In addition, false beaks (secondary conidiophores), unbranched, short, blunted, pale brown, (3.0–)5.3–16.0(–24.4) × (2.4–)2.8–4.1(–5.1) μm, (mean ± SD = 10.6 ± 5.4 × 3.4 ± 0.7 μm, n = 31). Conidial beakless mostly with a conical cell at the apex. Chlamydospores not observed.

**Figure 9.**
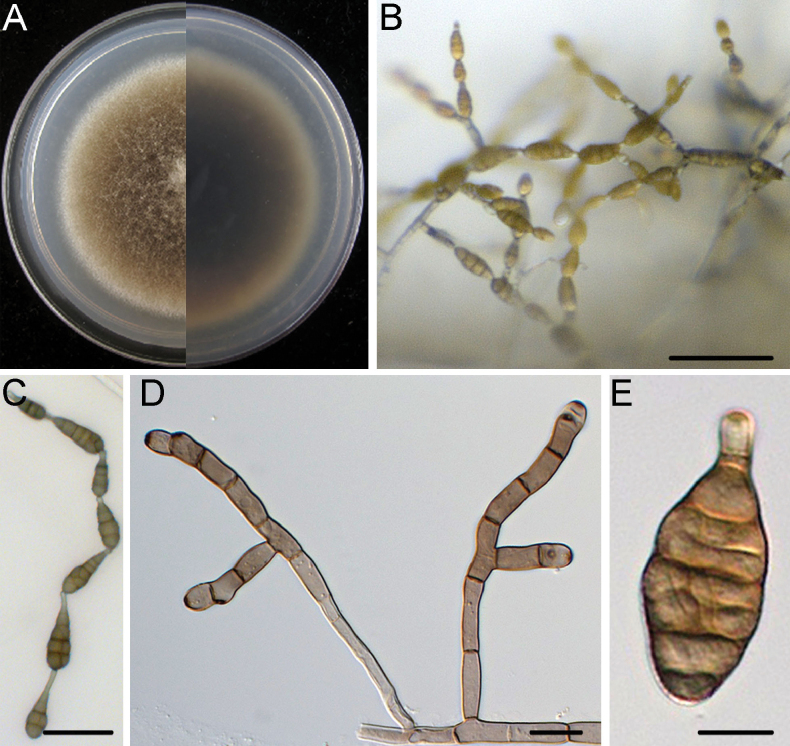
*Alternariaxinyangensis* (ZLS1) **A** colony on PCA after 6 days at 25 °C in the dark **B, C** sporulation patterns **D** conidiophores and conidiogenouse cells **E** conidium. Scale bars: 50 μm (**B, C**);10 μm (**D, E**).

##### Culture characteristics.

Colonies on PCA incubated at 25 °C in the dark growing at 7.2 mm/d; aerial hyphae cottony, olive green, with white margins; reverse centre black to greyish; sporulation abundant; diffusible pigment absent.

##### Additional materials examined.

China, Henan Province, Xinyang City, Zhenlei Mountain, 32°04'51"N, 114°07'23"E, isolated from leaf spots of *Cunninghamialanceolata*, May 2017, Wen-Li Cui, ZLS1-1, ZLS1-2, ZLS1-3, ZLS1-4; China, Henan Province, Xinyang City, Xinyang University, 32°08'20"N, 114°02'06"E, isolated from leaf spots of *C.lanceolata*, May 2017, Wen-Li Cui, XYXY06, XYXY8-2, XYXY15, XYXY15-1, XYXY15-2, XYXY15-3, XYXY15-4, XYXY16.

##### Notes.

The isolates of *A.xinyangensis* were phylogenetically close to *A.dongshanqiaoensis* (in this study, DSQ2-2), *A.citri* (ex-epitype, CBS 107.27), *A.cinerariae* (ex-epitype, CBS 612.72) and *A.kikuchiana* (ex-type, CBS 107.53) (Fig. 1). Between *A.xinyangensis* isolates and *A.dongshanqiaoensis* DSQ2-2, there were 1/453 differences in Alt a1, 1/510 in ITS, 8/664 in OPA10-2, 1/401 in endoPG, 1/757 in RPB2, 1/996 in SSU and 3/293 in TEF1. Between *A.xinyangensis* isolates and *A.citri* (ex-epitype, CBS 107.27), there were 1/453 differences in Alt a1, 3/510 in ITS, 8/664 in OPA10-2, 1/401 in endoPG, 1/996 in SSU and 3/293 in TEF1. Between *A.xinyangensis* isolates and *A.cinerariae* (ex-epitype, CBS 612.72), there were 1/453 differences in Alt a1, 3/510 in ITS, 8/664 in OPA10-2, 1/401 in endoPG, 1/996 in SSU and 3/293 in TEF1. Between *A.xinyangensis* isolates and *A.kikuchiana* (ex-type, CBS 107.53), there were 3/453 differences in Alt a1, 3/510 in ITS, 2/401 in endoPG, 1/757 in RPB2, 1/996 in SSU and 3/293 in TEF1. The PHI analysis showed that there was no significant recombination between *A.xinyangensis* isolates and their related species (Φ_w_ = 0.1647) (Fig. 2A). Distinguishing characteristics of this new species and other similar species of *Alternaria* spp. are shown in Table 2. Morphologically, conidial number in chains of the *A.xinyangensis* isolates were less than those of *A.dongshanqiaoensis* DSQ2-2 (2–7 conidia vs. 5–9 conidia). Conidia of the *A.xinyangensis* isolates were smaller than those of *A.citri*CBS 107.27 (ex-epitype) (19.9–31.8 × 8.6–12.9 μm vs. 25–40 × 15–25 μm) (Pierce 1902). Secondary conidiophores of the *A.xinyangensis* isolates were significantly shorter than those of *A.cinerariae*CBS 612.72 (ex-epitype) (5.3–16.0 × 2.8–4.1 μm vs. 80–159 × 5–9 μm) (Nishikawa and Nakashima 2020). Conidia in chains of the *A.xinyangensis* isolates were less than those of *A.kikuchiana*CBS 107.53 (ex-type) (2–7 conidia vs. 6–9 conidia) (Nishikawa and Nakashima 2019). In conclusion, the phylogenetic and morphological evidence supports this fungus as being a new species within the *Alternariaalternata* species complex.

### ﻿Pathogenicity assays

**Figure 10.**
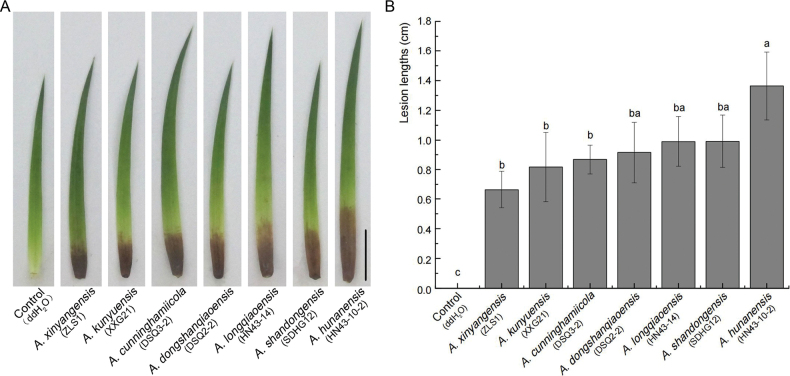
Symptoms on detached Chinese fir leaves **A** inoculated with isolates: *A.xinyangensis* (ZLS1), *A.kunyuensis* (XXG21), *A.cunninghamiicola* (DSQ3-2), *A.dongshanqiaoensis* (DSQ2-2), *A.longqiaoensis* (HN43-14), *A.shandongensis* (SDHG12) and *A.hunanensis* (HN43-10-2) **B** lesion length on detached Chinese fir leaves inoculated with *A.xinyangensis* (ZLS1), *A.kunyuensis* (XXG21), *A.cunninghamiicola* (DSQ3-2), *A.dongshanqiaoensis* (DSQ2-2), *A.longqiaoensis* (HN43-14), *A.shandongensis* (SDHG12) and *A.hunanensis* (HN43-10-2). Error bars represent standard error and different letters indicate significant difference, based on LSD’s range test at *P* < 0.05 (n = 12). Scale bar: 10 mm (**A**).

Pathogenicity was tested on detached Chinese fir leaves *in vitro* following Koch’s postulates for *A.xinyangensis* (ZLS1), *A.kunyuensis* (XXG21), *A.cunninghamiicola* (DSQ3-2), *A.dongshanqiaoensis* (DSQ2-2), *A.longqiaoensis* (HN43-14), *A.shandongensis* (SDHG12) and *A.hunanensis* (HN43-10-2). At five days’ post-inoculation, all the tested isolates caused leaf necrosis, with dark brown lesions. The control group remained symptom-less (Fig. 10A). After statistical analysis, these strains showed different levels of virulence. The virulence of *A.hunanensis* (HN43-10-2) was the strongest in all the *Alternaria* species studied, and its pathogenicity was significantly higher than those of *A.xinyangensis* (ZLS1), *A.kunyuensis* (XXG21) and *A.cunninghamiicola* (DSQ3-2) (P < 0.05), respectively, while there was no significant difference in pathogenicity amongst *A.xinyangensis* (ZLS1), *A.dongshanqiaoensis* (DSQ2-2), *A.shandongensis* (SDHG12), *A.kunyuensis* (XXG21), *A.longqiaoensis* (HN43-14) and *A.cunninghamiicola* (DSQ3-2) (P ≥ 0.05) (Fig. 10B).

The inoculated fungal isolates were re-isolated from the diseased spots on the inoculated leaves, but no fungus was isolated from the control leaves. Therefore, Koch’s postulates were satisfied and these isolates ZLS1, XXG21, DSQ3-2, DSQ2-2, HN43-14, SDHG12 and HN43-10-2 were determined to be the pathogens of leaf blight on *C.lanceolata*.

## ﻿Discussion

This study represents the first reports of leaf blight disease of Chinese fir in China caused by *Alternaria* spp. Phylogenetic analyses of the combined polylocus data set and morphological study showed that the 48 isolates obtained in this study grouped within Section Alternaria. It is surprising that the diversity of *Alternaria* species was so abundant in Chinese fir. It includes seven new species: *Alternariacunninghamiicola* sp. nov., *A.dongshanqiaoensis* sp. nov., *A.hunanensis* sp. nov., *A.kunyuensis* sp. nov., *A.longqiaoensis* sp. nov., *A.shandongensis* sp. nov. and *A.xinyangensis* sp. nov. The detached leaves of Chinese fir were selected for pathogenicity tests that confirmed the potential virulence. To our knowledge, it is the first comprehensive study on *Alternaria* species causing leaf blight disease on Chinese fir including diversity and pathogenicity of the pathogens.

Morphology was not the main means of identification, as *Alternaria* isolates could differ morphologically due to the different cultivating conditions and the overlap in the spore sizes of some species (Rahimloo and Ghosta 2015). Armitage et al. (2015) reported that the morphological characteristics used to delineate species in Alternariasect.Alternata are phenotypically similar and may vary amongst many morpho-species. These characteristics may be deceptive in the identification of these small-spored *Alternaria* species and would require stringent identification via phylogenetic studies (Kgatle et al. 2018). In this study, the single-locus phylogenies showed unclear resolution because of the limited number of informative sites per locus. For example, the SSU distinguishes *A.longqiaoensis* effectively with other species, but there is little resolution to distinguish between other species. The TEF1 gene could be informative for *A.xinyangensis*, *A.shandongensis*, and *A.kunyuensis* but not for *A.cunninghamiicola*, *A.dongshanqiaoensis*, *A.longqiaoensis* and *A.hunanensis*. In addition, it is also noted that the ITS region is a good phylogenetic marker, which could be informative for these isolates in this study, while LSU gene for distinguishing these isolates has a little effect. Perhaps these loci evolve at various rates and have different effective ways of evolution at several phylogenetic scales. For instance, Lawrence et al. (2013) reported that TEF1 and RPB2 are slow-evolving genes used to resolve early divergences in *Alternaria*, while Alt a1 is fast-evolving and can be used to infer evolutionary relationships at lower phylogenetic scales (Aung et al. 2020). Combined analyses of all nine loci are, thus, the major approach to identify *Alternaria* species.

A previous multi-locus phylogenetic study Woudenberg et al. (2013) established the taxonomic conclusions of morpho-species known under *A.alternata* based on the multi-locus phylogenetic analysis. Subsequently, Woudenberg et al. (2015) used the same analysis to determine the discrete lineages of Alternaria spp. in section Alternaria, which showed a 97–98% genomic similarity, concluding that species, such as *A.angustiovoide*, *A.citri*, *A.lini*, *A.mali* (CBS 106.24), *A.malvae* and *A.tenuissima* (CBS 918.96) did not make discrete groupings, but all are synonymous with *A.alternata* sensu stricto. Although Woudenberg et al. (2015) assigned 35 morpho-species as synonyms of *Alternariaalternata*, their affinities are still unclear due to inconsistencies, lack of morphological details and a comparison of single nucleotide polymorphisms. However, further studies, based on combined multi-locus phylogeny, showed that recent *A.alternata* species may not constitute a monophyletic group in DNA sequence-based phylogenies (Li et al. 2023). Morphological characters and phylogenetic analyses of the nine loci showed all 48 Alternaria isolates clustered in the Sect. Alternata in the phylogenetic tree and divide into seven distinct clusters in the current study. We compared these strains, based on morphology and phylogeny. Interestingly, our phylogenetic analyses show that the morpho-species of *A.alternata* can be separated into different clades and our novel taxa from Chinese fir are both morphologically and phylogenetically distinct from the *A.alternata* complex and other species in Alternariasect.Alternaria. Herein, based on these most recent classifications, these isolates from Chinese fir in this study are, thus, identified as the *A.alternata* complex including *A.cunninghamiicola*, *A.dongshanqiaoensis*, *A.hunanensis*, *A.kunyuensis*, *A.longqiaoensis*, *A.shandongensis* and *A.xinyangensis*.

The results of pathogenicity tests indicate that the seven new *Alternaria* species were pathogenic to Chinese fir. *Alternariahunanensis* exhibited the strongest virulence in the *Alternaria* species from the present study, and *A.xinyangensis*, *A.kunyuensis* and *A.cunninghamiicola* with weaker virulence especially in shoots of Chinese fir. Nevertheless, compared with our previous study, *Alternaria* species showing weaker virulence than those of *Colletotrichum* spp. (He et al. 2022) and *Fusarium* spp. (unpublished) and the results may explain why most of *Alternaria* species are facultative parasites and their pathogenicities are not too strong. *Alternaria* spp. may prefer to be saprobes or secondary pathogens growing in senescent, near-dead or dead plant tissues. The diseases caused by these pathogens often attack senescent and diseased leaves before crop maturity or when the growth of the hosts is poor. In addition, according to previous studies, some *Alternaria* taxa carry out facultative parasitism life cycles mainly depending on the following three aspects: damaging the cell walls of their hosts by mechanical penetration and the degrading enzymes, producing mycotoxins that target the cytoplasmic membrane, mitochondria, chloroplast and influencing the activity of enzymes related metabolisms, and mediating pathogenicity through signal transduction (Thomma 2003; Kang et al. 2013). At present, there are few studies on the pathogenic mechanism of *Alternaria* species, without revealing the specific process of host infection. Therefore, the thorough study of its pathogenic mechanism is the basis and key to solving the damage from *Alternaria*.

Until now, over 360 species of *Alternaria* are reported as plant pathogens and saprobes, resulting in the decline of forest quality and fruit decay during storage and resulting in huge economic losses (Wijayawardene et al. 2020; Li et al. 2023). For example, *A.citri* caused orange brown spot disease (Peever et al. 2004); *A.yali-inficiens* caused black spots of Japanese pear (Roberts 2005); *A.alternata*, *A.longipes* (Ellis & Everh.) E.W. Mason and *A.yali-inficiens* caused tobacco brown spots (Wang et al. 2018); *A.malicola* caused fruit spot on apple in China (Dang et al. 2018); *A.yunnanensis* Z.Y. Cai, X.Y. Liu, Y.X. Liu & Y.P. Shi caused foliage spots of rubber tree in China (Cai et al. 2019); *A.koreana* O. Hassan, B.B.N.D. Romain, J.S. Kim & T. Chang caused leaf spots of ovate-leaf *Atractylodes* in South Korea (Romain et al. 2022) and *A.capsicicola* Nasehi, Kadir & Abed-Asht. [nom. inval., Art. F.5.1 (Shenzhen)] caused leaf spots of pepper in Malaysia (Nasehi et al. 2014). Surprisingly, *A.alternata* had been considered as a saprobic fungus and to be nonpathogenic on Chinese cabbage (*Brassicarapa* L. pekinensis group) (Liu and Ke 1992; Zhang et al. 1998). However, *A.alternata* had been confirmed to be pathogenic on Chinese cabbage (Shi et al. 2021). In addition, many recent studies reported various diseases caused by *Alternaria* species. For example, Xiang et al. (2023) reported the black spots caused by *A.alternata* on persimmon fruit in China. Yan et al. (2023) identified *A.tenuissima* causing leaf spots on *Loniceracaerulea* L. in Heilongjiang Province, China. Zhou et al. (2023) characterised *A.alstroemeriae* E.G. Simmons & C.F. Hill, a causal agent of grey spots on tobacco in China. Dantes et al. (2022) discovered *A.cinerariae* causing leaf blight on *Farfugiumjaponicum* (L.) Kitam. in South Carolina, USA. To our knowledge, however, so far, there is no detailed record that *Alternaria* spp. have been identified as pathogens on Chinese fir, except *Alternaria* sp. reported by Anonymous (1976).

In summary, our study provides the first systematic and polyphasic study from morphological, molecular and pathogenicity aspects to study *Alternaria* spp. associated with Chinese fir and reports seven novel species, *A.cunninghamiicola*, *A.dongshanqiaoensis*, *A.hunanensis*, *A.kunyuensis*, *A.longqiaoensis*, *A.shandongensis* and *A.xinyangensis* causing leaf blight on Chinese fir. However, more studies are necessary on these new taxa in order to elucidate their host range, specificity, mechanism of infection, and global distribution, as well as their potential impact on the Chinese fir industry.

## Supplementary Material

XML Treatment for Alternaria cunninghamiicola

XML Treatment for Alternaria dongshanqiaoensis

XML Treatment for Alternaria hunanensis

XML Treatment for Alternaria kunyuensis

XML Treatment for Alternaria longqiaoensis

XML Treatment for Alternaria shandongensis

XML Treatment for Alternaria xinyangensis

## ﻿Additional information

### Author ORCIDs

Jiao He https://orcid.org/0000-0002-4146-2223

De-Wei Li https://orcid.org/0000-0002-2788-7938

Wen-Li Cui https://orcid.org/0009-0005-7515-7672

Lin Huang https://orcid.org/0000-0001-7536-0914

### Data availability

All of the data that support the findings of this study are available in the main text or Supplementary Information.

## Supplementary materials

Supplementary material 1 Supplementary information Data type docx Explanation note **table S1.** Fungal cultures isolated from Chinese fir in this study. **table S2.** Primers used for PCR amplification and DNA sequences. https://binary.pensoft.net/file/962743This dataset is made available under the Open Database License (http://opendatacommons.org/licenses/odbl/1.0/). The Open Database License (ODbL) is a license agreement intended to allow users to freely share, modify, and use this Dataset while maintaining this same freedom for others, provided that the original source and author(s) are credited.Jiao He, De-Wei Li, Wen-Li Cui, Lin Huang
