# Supplementary material for: ﻿Seven new species of Alternaria (Pleosporales, Pleosporaceae) associated with Chinese fir, based on morphological and molecular evidence
Source: MycoKeys. 2024 Jan 5;101:1–44. doi: 10.3897/mycokeys.101.115370 (PMC10787357; doi:10.3897/mycokeys.101.115370)
Supplement: Supplementary material 1 — Supplementary information [file mycokeys-101-001-s001.docx]

# Supplementary Materials

1273

**Supplementary Table 1.** Fungal cultures isolated from Chinese fir in this study.

| **Strian code** | **Location** | **Longitude** | **Latitude** | **Numbers** |
| --- | --- | --- | --- | --- |
| ZLS1 ZLS1-1 | Zhenlei Mountain, Xinyang |  |  |  |
|  | | | | |
| ZLS1-2 City, Henan Province 114°07′23″ 32°04′51″ 5 | | | | |
| ZLS1-3 | | | | |
| ZLS1-4 | | | | |
| XYXY06 XYXY15 XYXY8-2 |  |  |  |  |
|  | | | | |
|  | | | | |
| XYXY16 Xinyang University, Xinyang | | | | |
| XYXY15-1 City, Henan Province 114°02′06″ 32°08′20″ 8 | | | | |
| XYXY15-2 | | | | |
| XYXY15-3 | | | | |
| XYXY15-4 | | | | |
| XXG21 XXG22 |  |  |  |  |
|  | | | | |
| XXG26-2 Kunyu Mountain, Yantai City, | | | | |
| XXG31 Shandong Province 121°46′05″ 37°15′22″ 6 | | | | |
| XXG30 | | | | |
| XXG12-2 | | | | |
| DSQ3-2 DSQ2-2 DSQ3-2-1 |  |  |  |  |
|  | | | | |
|  | | | | |
| DSQ3-2-2 | | | | |
| DSQ3-2-3 Dongshanqiao Forest Farm, 118°46′12″ 31°51′11″ 9  Nanjing City, Jiangsu Province | | | | |
| DSQ3-2-4 | | | | |
| DSQ2-2-1 | | | | |
| DSQ2-2-2 | | | | |
| DSQ2-2-3 | | | | |
| HN43-6-1 HN43-6-1-1 HN43-6-1-2 |  |  |  |  |
|  | | | | |
|  | | | | |
| HN43-6-1-3 | | | | |
| HN43-6-1-4 Longqiao Town, Yiyang City, 112°29′7″ 28°27′24″ 14  Hunan Province | | | | |
| HN43-10-2 | | | | |
| HN43-10-2-1 | | | | |
| HN43-10-2-2 | | | | |
| HN43-10-2-3 | | | | |

| **Strian code** | **Location** | **Longitude** | **Latitude** | **Numbers** |
| --- | --- | --- | --- | --- |
| HN43-10-2-4 | | | | |
| HN43-14 | | | | |
| HN43-14-1 | | | | |
| HN43-14-2 | | | | |
| HN43-14-3 | | | | |
| SDHG12 SDHG12-1 | Hougou village, Penglai City, |  |  |  |
|  | | | | |
| SDHG12-2 Shandong Province 120°46'48″ 37°27'32″ 5 | | | | |
| SDHG12-3 | | | | |
| SDHG12-4 | | | | |
| LY15 | Lianfeng Town, Longyan City, Fujian Province | 117°01′50″ | 25°09′27″ | 1 |
| Total |  |  |  | 48 |

1274

1275

**Supplementary Table 2.** Primers used for PCR amplification and DNA sequences.

| Genes/Regions1 | Primer | (5'-3') Primer sequences2 |
| --- | --- | --- |
| TEF1 | EF983 | GCYCCYGGHCAYCGTGAYTTYAT |
|  | 2218R | ATGACACCRACRGCRACRGTYTG |
| GAPDH | GDF-1 | GCCGTCAACGACCCCTTCATTGA |
|  | GDR-1 | GGGTGGAGTCGTACTTGAGCATGT |
| LSU | LR5 | TCCTGAGGGAAACTYCG |
|  | LROR | ACCCGCTGAACTYAAGC |
| ITS | ITS1 | TCCGTAGGTGAACCTGCGG |
|  | ITS4 | TCCTCCGCTTATTGATATGC |
| SSU | NS1 | GTAGTCATATGCTTGTCTC |
|  | NS4 | CTTCCGTCAATTCCTTTAAG |
| RPB2 | 5f2 | GGGGTGATCAGAAGAAGGC |
|  | 7cr | CCCATGGCTTGTTTGCCCAT |
| Alt a 1 | Alt-al-for | ATGCAGTTCACCACCATCGC |
|  | Alt-a1-rev | ACGAGGGTGAYGTAGGCGTC |
| endoPG | PG3 | TACCATGGTTCTTTCCGA |
|  | PG2b | GAGAATTCRCARTCRTCYTGRTT |
| OPA10-2 | OPA 10-2R | GATTCGCAGCAGGGAAACTA |
|  | OPA 10-2L | TCGCAGTAAGACACATTCTACG |
| 1 TEF1: Translation elongation factor 1-alpha; GAPDH: Glyceraldehyde-3-phosphate dehydrogenase; LSU: The 28S nrDNA; ITS: Internal transcribed spacer regions of the rDNA and 5.8S region; SSU: The 18S nrDNA; RPB2: RNA polymerase second largest subunit; Alt a 1: Alternaria major allergen gene; endoPG: Endopolygalacturonase; OPA10-2: An anonymous gene region. | | |
| 2 Y = C or T; H = A, G or T; R = A or G. | | |
